# Horizontal-Acquisition of a Promiscuous Peptidoglycan-Recycling Enzyme Enables Aphids To Influence Symbiont Cell Wall Metabolism

**DOI:** 10.1128/mBio.02636-21

**Published:** 2021-12-21

**Authors:** Thomas E. Smith, Mijoon Lee, Maria D. Person, Dusan Hesek, Shahriar Mobashery, Nancy A. Moran

**Affiliations:** a Department of Integrative Biology, University of Texas at Austin, Austin, Texas, USA; b Department of Chemistry and Biochemistry, University of Notre Dame, Notre Dame, Indiana, USA; c Mass Spectrometry and Proteomics Facility, University of Notre Dame, Notre Dame, Indiana, USA; d Center for Biomedical Research Support Proteomics Facility, University of Texas at Austin, Austin, Texas, USA; University of Connecticut

**Keywords:** *Buchnera*, carboxypeptidase, cell wall, endopeptidase, enzyme promiscuity, horizontal gene transfer, multifunctional, pea aphid, peptidoglycan, symbiosis

## Abstract

During evolution, enzymes can undergo shifts in preferred substrates or in catalytic activities. An intriguing question is how enzyme function changes following horizontal gene transfer, especially for bacterial genes that have moved to animal genomes. Some insects have acquired genes that encode enzymes for the biosynthesis of bacterial cell wall components and that appear to function to support or control their obligate endosymbiotic bacteria. In aphids, the bacterial endosymbiont Buchnera aphidicola provides essential amino acids for aphid hosts but lacks most genes for remodeling of the bacterial cell wall. The aphid genome has acquired seven genes with putative functions in cell wall metabolism that are primarily expressed in the aphid cells harboring *Buchnera*. In analyses of aphid homogenates, we detected peptidoglycan (PGN) muropeptides indicative of the reactions of PGN hydrolases encoded by horizontally acquired aphid genes but not by *Buchnera* genes. We produced one such host enzyme, *Ap*LdcA, and characterized its activity with both cell wall derived and synthetic PGN. Both *Ap*LdcA and the homologous enzyme in Escherichia coli, which functions as an l,d-carboxypeptidase in the cytoplasmic PGN recycling pathway, exhibit turnover of PGN substrates containing stem pentapeptides and cross-linkages via l,d-endopeptidase activity, consistent with a potential role in cell wall remodeling. Our results suggest that *Ap*LdcA derives its functions from the promiscuous activities of an ancestral LdcA enzyme, whose acquisition by the aphid genome may have enabled hosts to influence *Buchnera* cell wall metabolism as a means to control symbiont growth and division.

## INTRODUCTION

The near-universal ability of enzymes to perform promiscuous reactions is increasingly recognized as a starting point in the evolution of novel functions (1). Promiscuous activities become functional when, in the context of a new environment and/or mutation(s), they contribute to fitness, often via complementation of metabolic inadequacies (2–4). Host-associated bacteria that have experienced extreme genome reduction often lack essential genes or whole pathways (5), such that multifunctional enzymes derived from ancestrally promiscuous enzymes have been suggested as a likely means of compensation (6, 7). This idea is supported by examples in multiple bacterial lineages, including the mammalian pathogen Chlamydia (8, 9) and insect-associated *Wolbachia* (10) and Buchnera aphidicola (11) symbionts.

Alternatively, host genomes may acquire genes via horizontal-gene transfer (HGT) to supplement symbiont shortcomings. While HGT is relatively rare in eukaryotes, it occurs most often from host-associated bacteria (12) and has proven instrumental in eukaryotic evolution (13–16), most notably in the context of mitochondrial and plastid evolution (17, 18). Among insect symbioses, horizontally transferred genes (HTGs) appear to provide hosts with novel functions and, in some cases, may improve their ability to regulate or benefit from symbionts (12, 19). Recently, the compensatory nature of several mealybug HTGs has been revealed—the insect genome encodes enzymes of the peptidoglycan (PGN) synthesis pathway that symbionts lack (20), and these proteins localize within symbionts and participate in cell wall construction (21).

**FIG 1:**
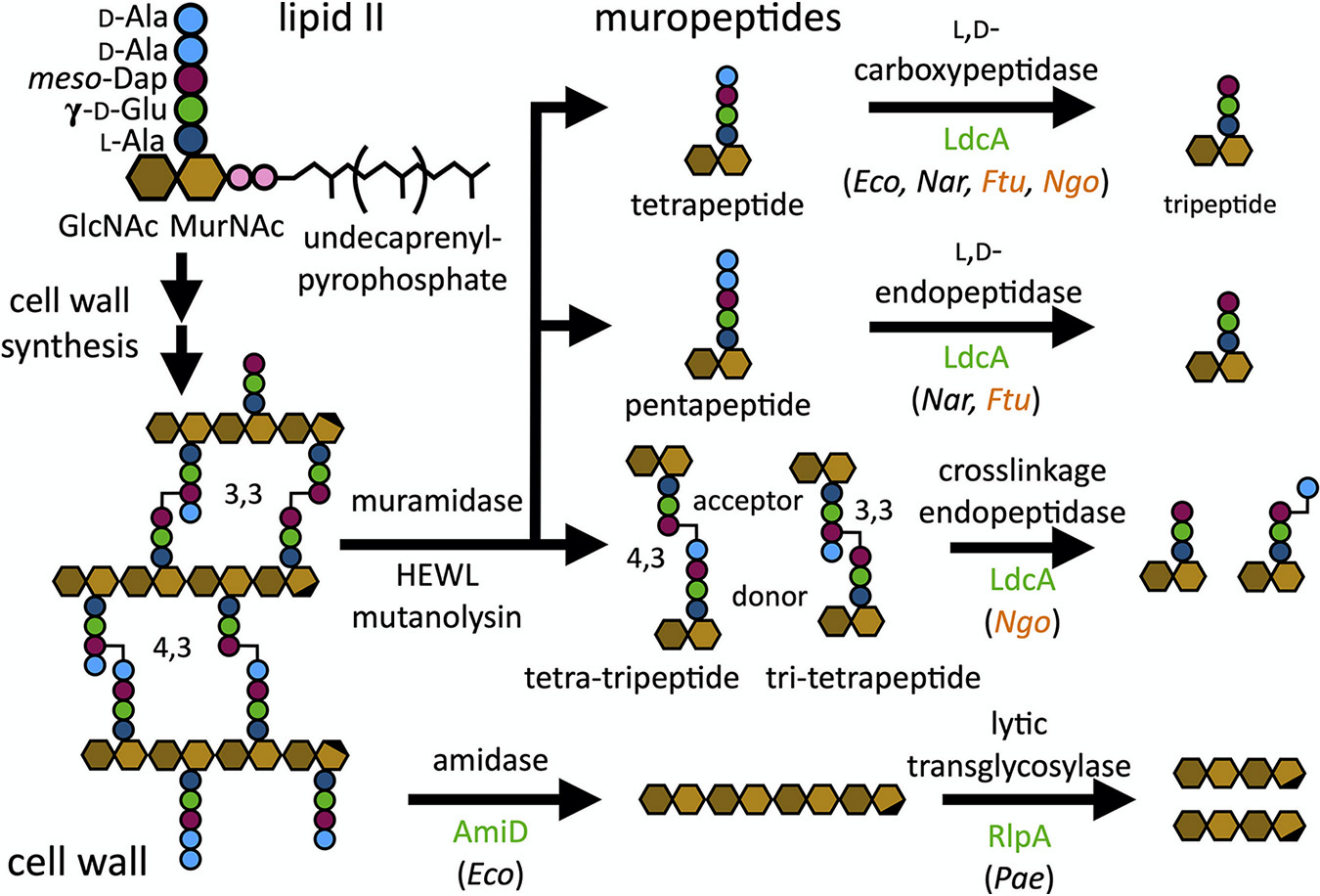
Schematic representation of the enzymatic activites of previously characterized homologs of the aphid LdcA, AmiD, and RlpA enzymes toward peptidoglycan (PGN). The cell wall is assembled from lipid II, consisting of *N-*acetylglucosamine (GlcNAc), *N*-acetylmuramic acid (MurNAc), l-alanine, γ-d-glutamate, *meso*-diaminopimelic acid (Dap), and d-alanine. Cell wall digestion with muramidase produces muropeptides, many of which are suitable substrates for known LdcA enzyme activities, while AmiD and RlpA act on the polymeric cell wall. Enzyme reactions appear above reaction arrows, while enzyme names are shown below. Enzymes shown in green indicate those for which aphid-encoded homologs exist. Organism abbreviations, shown in parentheses, denote the species for which the reaction has been demonstrated for the enzyme homolog: *Eco*, E. coli (27, 67); *Nar*, Novosphingobium aromaticivorans (31); *Ftu*, *Francisella tularensis* (29); *Ngo*, Neisseria gonorrhoeae (30); and *Pae*, Pseudomonas aeruginosa (68). Organism abbreviations shown in orange designate enzymes capable of utilizing both muropeptides and cell wall as substrates.

Aphids require *Buchnera* symbionts to provide essential amino acids that are missing from their exclusive diet of phloem sap. Interestingly, the pea aphid (*Acyrthosiphon pisum*) genome contains eight HTGs with putative functions in PGN metabolism (Fig. 1) (22, 23). Seven of these aphid HTGs appear important for symbiosis based on their increased expression in bacteriocytes (23), the specialized host cells where *Buchnera* reside, relative to other host tissues. In addition, RNAi knockdown of HTG expression reduces *Buchnera* abundance (24), and HTG expression is correlated with aphid genotypes displaying high symbiont abundances (25). Considering the close coordination of PGN metabolism with cell growth and division machinery in bacteria (26), these observations suggest that host PGN enzymes may play a role in regulating *Buchnera* proliferation.

Of the seven aphid HTGs implicated in symbiosis, all but one putatively function in cell wall remodeling. This gene, *ldcA*, encodes a homolog of the l,d-carboxypeptidase (*Ap*LdcA) involved in PGN recycling (27), a cytoplasmic process that is absent in *Buchnera* but present in free-living bacteria like Escherichia coli, a close relative of *Buchnera* (Fig. 1) (28). While E. coli LdcA (*Ec*LdcA) is known to utilize only solubilized PGN fragments (muropeptides), LdcA homologs from some intracellular pathogens are exported to the periplasm and display a shift in substrate tolerance, modifying the polymeric cell wall in addition to soluble muropeptides (29, 30). Furthermore, LdcA homologs exhibit multifunctionality, demonstrating endopeptidase activities in addition to their carboxypeptidase function (29–31). We hypothesized that *Ec*LdcA might display a similar shift in activity that could enable aphids to control or support its symbionts. Specifically, endopeptidases are essential for E. coli because they are required to make space for the insertion of nascent PGN strands into the cell wall (32)—an endopeptidase may be required by *Buchnera* but encoded by the host. If *Ap*LdcA is an endopeptidase, this novel function may derive from a promiscuous enzyme activity present in the ancestral enzyme.

In the present work, we investigated the hypothesis that *Ap*LdcA displays key differences from a free-living bacterial homolog, *Ec*LdcA. First, we provide evidence that PGN hydrolases, including an endopeptidase, are active in the aphid-*Buchnera* system, producing muropeptides that can be isolated from the aphid hemolymph indicative of their physiological relevance. Second, we demonstrate that *Ap*LdcA retains its l,d-carboxypeptidase function toward soluble muropeptides and also exhibits l,d-endopeptidase activity against both stem pentapeptides and cross-linked peptidoglycan. This feature likely derives from ancestral enzyme promiscuity, since the closely related *Ec*LdcA is also capable, albeit to a lesser extent, of l,d-endopeptidase activity. Taken together, these results reveal potential host adaptations that have capitalized on the catalytic and substrate promiscuity of LdcA in order to target symbiont PGN.

## RESULTS

### Complex PGN can be isolated from whole aphids.

We first sought to understand the role that host HTGs may play in *Buchnera* PGN metabolism by characterizing *Buchnera*’s cell wall. Cell wall PGN is comprised of repeating units of β-1,4-linked *N-*acetylglucosamine (GlcNAc) and *N*-acetylmuramic acid (MurNAc) disaccharide with a short stem peptide attached to the MurNAc lactyl moiety via amide bond (33). In Gram-negative bacteria, the stem peptide typically consists of five amino acids: l-Ala, γ-d-glutamate, *meso*-diaminopimelic acid (*m*Dap), d-Ala, and d-Ala (pentapeptide). Synthesis of PGN, the major constituent of the cell wall (also called murein), begins in the cytoplasm with the multistep construction of lipid II and transitions to the periplasm where the cell wall, or murein sacculus, is assembled from lipid II by a series of additional enzymes (33). Cell wall remodeling is necessary for bacterial growth and division, for antibiotic resistance, for repair of the cell wall, and for the insertion of outer membrane proteins (34, 35). Bacteria additionally recycle PGN by importing muropeptides, the products of cell wall remodeling events, into the cytoplasm to be shunted back into lipid II biosynthesis (35). A wide range of chemical modifications are possible during these processes, leading to distinct cell wall compositions among even closely related bacterial species (34–36). The *Buchnera* genome has a greatly reduced repertoire of cell wall synthesis and remodeling genes relative to E. coli, including a lack of any typical carboxypeptidases or cross-linkage endopeptidases. We thus anticipated that evidence of these enzyme activities, such as trimmed stem peptides, might be absent or limited in the *Buchnera* cell wall. *Buchnera* is not culturable outside its aphid host, but we were able to purify PGN directly from aphids. We subjected *A. pisum* homogenate to high-performance liquid chromatography (HPLC) and screened fractions for muropeptides using nano ultrahigh performance liquid chromatography-tandem mass spectrometry (UPLC-MS/MS) and a novel proteomics-based approach for the automated identification of muropeptides (37).

**FIG 2:**
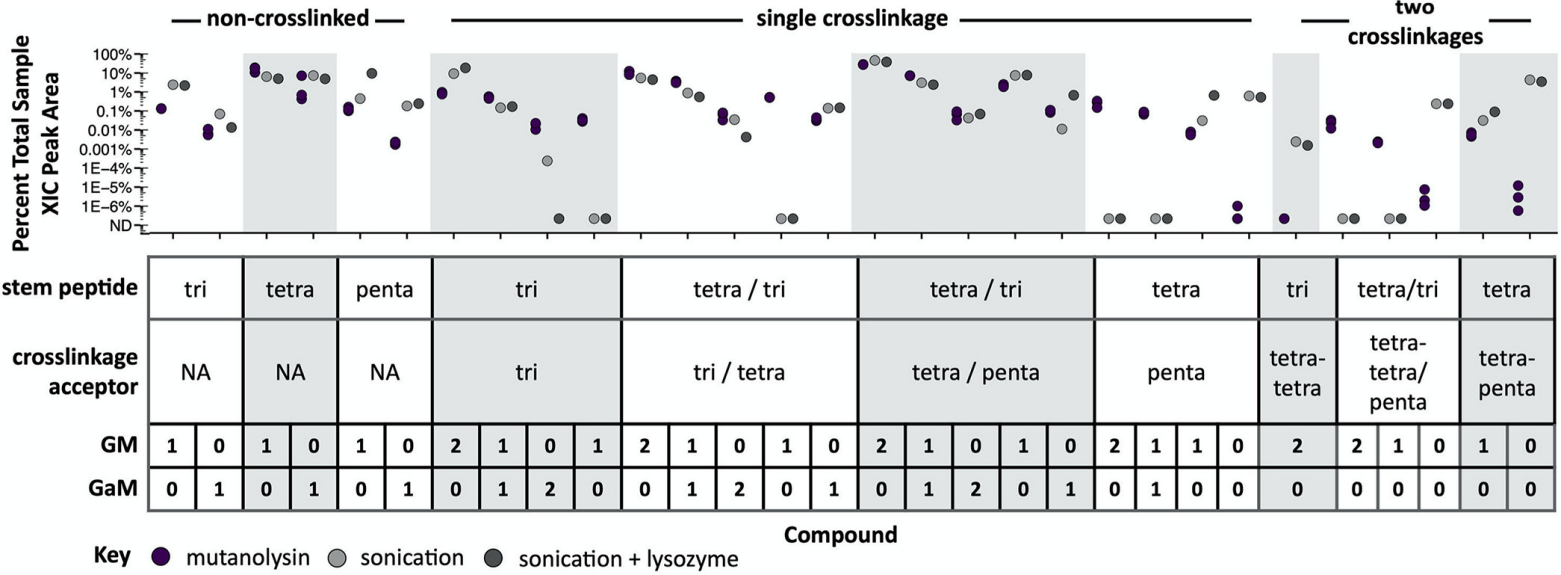
Composition of *Buchnera*-derived cell wall fragments purified from *A. pisum*. Aphid homogenate was successively filtered to components of ≤10 kDa and subjected to LC-MS/MS analysis. Muropeptide compounds were identified from MS/MS spectra and extracted-ion chromatogram (XIC) peak areas determined using Byonic and Byologic softwares, respectively (Protein Metrics). Areas were baseline subtracted and normalized by sample, such that the data shown is the percentage of total PGN represented by each compound. Untreated mutanolysin-derived E. coli muropeptides are shown for comparison (purple). Two treatments were used without replicates: aphid homogenate was sonicated to lyse *Buchnera* cells (light gray) or additionally treated with lysozyme to digest *Buchnera* cell walls (dark gray). The table describes the compound structure: PGN compounds vary by stem peptide sequence and glycan (GM = GlcNAc-MurNAc, GaM = GlcNAc-anhydro-MurNAc). For distinct compounds that are equivalent in mass (differing either in stem peptide sequence or cross-linkage type), we were unable to quantify each compound abundance independently, because the two compounds could not be chromatographically resolved—such structural isomers were integrated together, and their sequences are reported with variable residues shown separated by backslashes within parentheses, such that the same relative position within parentheses refers to the sequence of one isomer.

To compare *Buchnera* PGN with that of its closest free-living relative, we also analyzed E. coli PGN derived from digestion of the isolated sacculus with the muramidase mutanolysin. The E. coli cell wall exhibits a range of chemical modifications, including GlcNAc-anhydro-MurNAc (G-aM) disaccharides at the termini of PGN strands, stem peptide cross-linkages that give the cell wall its mesh-like architecture, and a low level of substitution of stem peptide d-Ala residues for noncanonical l- or d-amino acids (NCLAAs and NCDAAs, respectively). We detected a similarly complex assortment of muropeptides from aphids with both GlcNAc-MurNAc (GM) and G-aM glycans, variable stem peptide lengths and sequences, and diverse cross-linked compounds, including those derived from three strands of peptidoglycan (Fig. 2; see also Fig. S1A and B in the supplemental material). We analyzed fractions from two distinct homogenate treatments—both were sonicated to lyse *Buchnera* cells, while one was additionally treated with hen egg-white lysozyme (HEWL) to digest the cell wall. Untreated and lysozyme-treated samples had largely similar PGN profiles and sonication alone is insufficient to shear glycan chains into small units (38), suggesting that soluble muropeptides are produced in aphids in the absence of exogenously added lysozyme.

**FIG 3:**
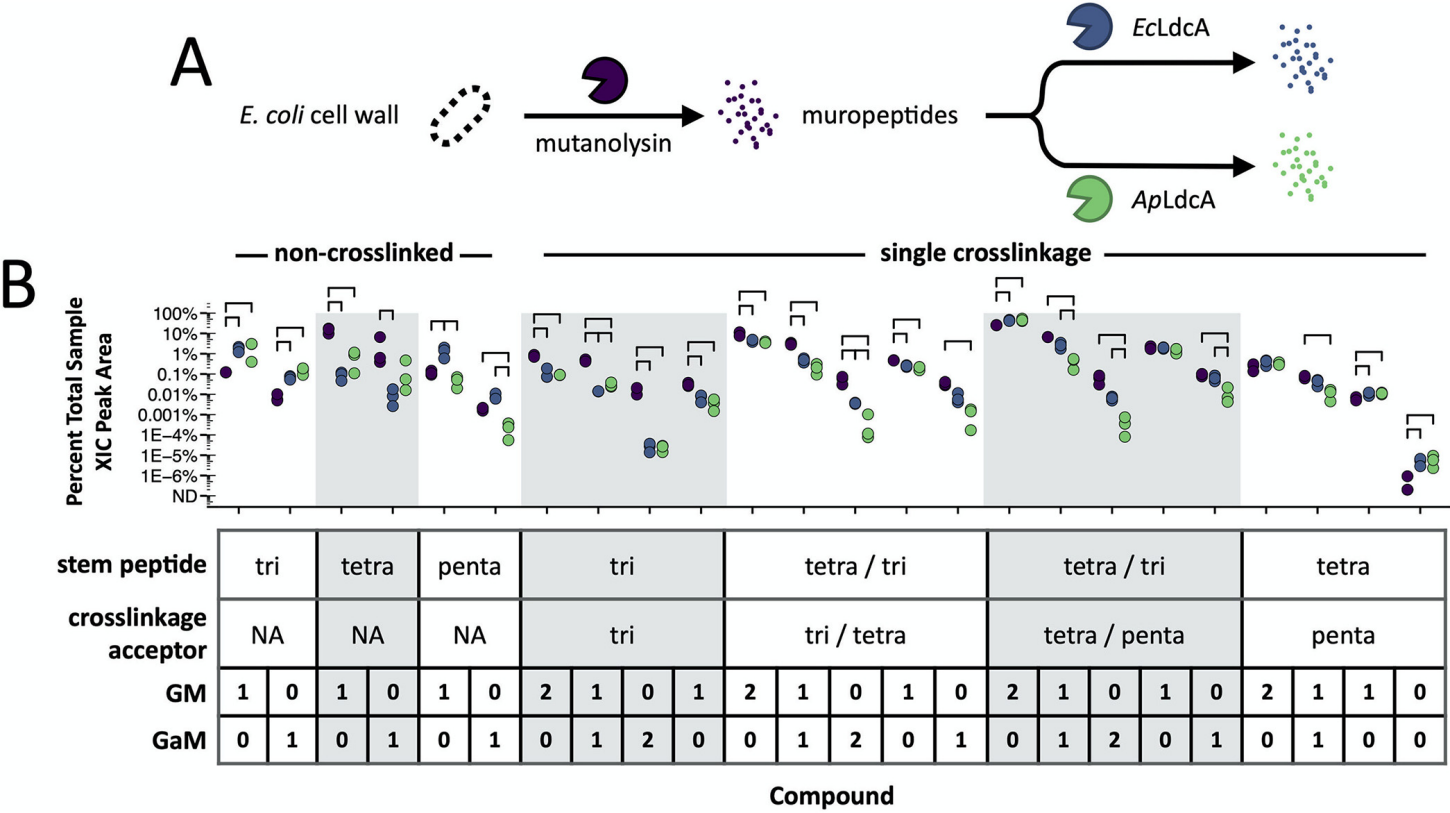
Comparison of the muropeptide composition and abundance of soluble PGN substrates following treatment with *Ec*LdcA and *Ap*LdcA. (A) Mutanolysin-derived E. coli muropeptides were treated with *Ec*LdcA and *Ap*LdcA and subjected to the same LC-MS/MS analysis used for the data in Fig. 2. (B) Areas were baseline subtracted and normalized by sample. For each compound, the data shown are the average percentages of total PGN from three replicates (see Table S1). Comparisons between treatments were made using Tukey’s HSD test with adjustment for false discovery. All bars shown represent *P* values of <0.05.

10.1128/mBio.02636-21.2 FIG S1 Composition of *Buchnera-*derived and LdcA-treated E. coli muropeptides as determined by LC-MS/MS. (A and B) Noncanonical non-cross-linked (A) and singly cross-linked (B) muropeptides identified from *A. pisum* homogenate (Fig. 2). Untreated E. coli mutanolysin-derived muropeptides are shown for comparison (purple). Two treatments were used without replicates: aphid homogenate was either sonicated to lyse *Buchnera* cells (light gray) or sonicated and additionally treated with lysozyme to digest *Buchnera* cell walls (dark gray). (C and D) Protein fractions generated during IMAC purification of recombinantly expressed *A. pisum* LdcA (C) and E. coli LdcA (D). For each SDS-PAGE gel, fractions analyzed included the insoluble pellet (P), soluble cell lysate (L), Ni-NTA flowthrough (FT), lysis buffer wash (W1), HEPES buffer wash (W2), and HEPES-buffered elutions of increasing imidazole concentrations: 50 mM (E1), 100 mM (E2), 200 mM (E3), and 500 mM (E4). (E to G) Noncanonical non-cross-linked (E), noncanonical singly cross-linked (F), and canonical doubly cross-linked (G) muropeptides after treatment of mutanolysin-derived E. coli muropeptides (purple) with *Ec*LdcA (blue) and *Ap*LdcA (green) (Fig. 3). All stem peptide sequences consist of l-Ala, γ-d-Glu, and *meso*-l-Dap, followed by the single-letter amino acid code indicated in the compound table. The absence of an amino acid indicates that the tripeptide is the complete stem-peptide sequence. Glycan substituents include GlcNAc-MurNAc (GM) and GlcNAc-anhydro-MurNAc (GaM). Download FIG S1, TIF file, 2.6 MB. Copyright © 2021 Smith et al. 2021 Smith et al. https://creativecommons.org/licenses/by/4.0/ This content is distributed under the terms of the Creative Commons Attribution 4.0 International license.

The composition of muropeptides in aphid homogenate implicates several host and/or symbiont PGN enzymes in their origin. Muropeptides containing terminal G-aM (including G-aM itself) are products of lytic transglycosylases such as *Ap*RlpA (Fig. 1) or *Buchnera*’s MltA and MltE enzymes, nonhydrolytic enzymes that fragment PGN chains (35, 39). On the other hand, GM-substituted muropeptides could be produced by hydrolytic muramidases, like the two endogenous invertebrate (i-type) lysozymes (NCBI gene IDs 100160909 and 100168424) that are more highly expressed in bacteriocytes relative to other host tissues (40). We detected muropeptides containing tripeptide and tetrapeptide stems, which are likely derived from carboxypeptidase and/or endopeptidase activities (Fig. 1). Since the *Buchnera* genome encodes no recognizable l,d-carboxypeptidases or endopeptidases and LdcA homologs from intracellular pathogens collectively exhibit both of these functions (29, 30), it is possible that *Ap*LdcA could be responsible for producing these stem peptides in *Buchnera*.

Aphid PGN also includes cross-linked PGN compounds containing 4,3- and 3,3-cross-linkages (Fig. 1). The 4,3-cross-linkages, which predominate in E. coli, are likely formed by the d,d-transpeptidases PBP1B and PBP3 encoded by *Buchnera* during cell wall synthesis, but 3,3-cross-linkages typically require the l,d-transpeptidases YnhG and YcbB (41), which *Buchnera* spp. lack. Because some cross-linked muropeptides are equivalent in mass and yet display distinct cross-linkage types (i.e., 4,3-cross-linked tetra-tripeptide and 3,3-cross-linked tri-tetrapeptide), these could not be definitively distinguished by their MS/MS fragmentation patterns, as described by Bern et al. (37). However, the presence of tri-tripeptide cross-linked stems, which contain only 3,3-cross-linkages, indicates that both 4,3- and 3,3-cross-linkage types are represented among PGNs derived from aphid homogenate (Fig. 2). In addition, while E. coli PGN is devoid of cross-linked stem peptides lacking any glycan substituents, we observed glycanless cross-linked stem peptides among muropeptides from aphid homogenate that are likely products of *Ap*AmiD or *Buchnera*’s AmiB amidase (Fig. 2).

Stem peptides may include noncanonical residues in place of one or both terminal d-Ala residues. NCDAAs are introduced to stem peptides either during lipid II synthesis via racemase enzymes or during cell wall synthesis via l,d-transpeptidases (42) and play a role in regulating PGN composition (42–44). NCLAAs found within stem peptides derive from covalent attachment of outer membrane proteins, such as murein lipoprotein (Lpp) in E. coli (45) and can be detected following proteolytic digest (46). Though our approach is incapable of discerning amino-acid stereochemistry, we observed that 33.1% of all E. coli detected muropeptides contain atypical amino acids, which are present in both non-cross-linked (see Fig. S1A) or cross-linked stem peptides (see Fig. S1B and Table S1). Some noncanonical stem peptides reach proportions similar to that of canonical tri- or pentapeptides. Among these, AE-*m*Dap-K and AE-*m*Dap-KR stem peptides are derived from Lpp. In contrast, only 10.7% of aphid muropeptides contain noncanonical amino acids, and a much lower diversity of stem sequences is represented. Despite this, we detected AE-*m*Dap-K and AE-*m*Dap-KR stems in aphids (see Fig. S1A and B). This observation is unexpected for two reasons: (i) the *Buchnera* genome lacks the gene encoding Lpp, as well as any homologs of the three l,d-transpeptidases, in E. coli that cross-links Lpp to PGN (*ldtA* to *ldtC*) (41), and (ii) unlike the E. coli sacculus, aphid PGN samples were not treated with proteases. Most Gram-negative bacteria lack Lpp homologs—in these species, the cell wall is covalently linked to different outer membrane proteins (46), suggesting the same may be true for *Buchnera*. While the source of AE-*m*Dap-KR muropeptides in aphid homogenate is unclear, these molecules are abundant and are likely part of a specific and significant process in *Buchnera*.

10.1128/mBio.02636-21.8 TABLE S1 Differences in average percent abundance of total PGN per compound between mutanolysin-digest of E. coli sacculus (E. coli), muropeptides isolated from *A. pisum* homogenate (*Buchnera*), and E. coli PGN treated with *Ec*LdcA (*Ec*LdcA) and *Ap*LdcA (*Ap*LdcA). Download Table S1, DOCX file, 0.07 MB. Copyright © 2021 Smith et al. 2021 Smith et al. https://creativecommons.org/licenses/by/4.0/ This content is distributed under the terms of the Creative Commons Attribution 4.0 International license.

Soluble muropeptides purified from aphid homogenate are derived from the *Buchnera* cell wall and reflect the collective enzymatic activities of both (i) cell wall remodeling and (ii) any downstream processing of soluble remodeling products. Our results show that endopeptidase and l,d-carboxypeptidase activities are involved in one or both of these processes. Furthermore, we found that *Buchnera* PGN is essentially as complex as that of E. coli, notwithstanding the limited set of PGN enzymes. This level of complexity also suggests that *Buchnera* muropeptides are not exhaustively degraded by symbiont or host PGN enzymes, indicating that these enzymes may play a more specific role in sculpting the *Buchnera* cell wall architecture.

### Characterization of LdcA activities using a multisubstrate, automated assay.

Next, we investigated the reactions of *Ap*LdcA and *Ec*LdcA *in vitro*. We expressed the *ldcA* genes in E. coli and purified recombinant *Ap*LdcA and *Ec*LdcA proteins by immobilized metal-affinity chromatography (IMAC) (see Fig. S1C and D). We then treated muropeptides derived from mutanolysin digestion of E. coli cell walls with the recombinant proteins (Fig. 3A). These compounds are similar to the substrates normally encountered by *Ec*LdcA in the cytoplasm, consisting of at most a single glycan substituent per stem peptide. In addition, the complexity of E. coli PGN in terms of the abundance and diversity of cell wall modifications allows for simultaneous evaluation of a wide range of potential enzyme substrates. Treated muropeptides were reduced with sodium borohydride and desalted by HPLC before applying the same proteomics-based approach used above to identify individual muropeptide compounds and evaluate LdcA activity toward each potential substrate (Fig. 3B). The same quantity of an identical preparation of E. coli sacculus was used for each reaction, such that, for a given compound, relative differences in abundance between treatments are essentially quantitative (see Table S1).

Relative to untreated E. coli muropeptides, we observed reduced tetrapeptide abundance for both *Ec*LdcA and *Ap*LdcA with concurrent increases in the amount of tripeptides, demonstrating that the l,d-carboxypeptidase activity of these enzymes can be readily detected by our approach as a decrease in substrate and an accumulation of product (Fig. 3B; see also Table S1). Interestingly, we observed a decrease in pentapeptide abundance for *Ap*LdcA and an increase for *Ec*LdcA. The former suggests that *Ap*LdcA acts as an l,d-endopeptidase that converts pentapeptides directly to tripeptides, an activity previously reported for LdcA homologs from Novosphingobium aromaticivorans and Francisella tularensis (29, 31). An explanation for the latter observation is described in the following section. We also detected a lower abundance of several noncanonical tetrapeptide monomers for both enzymes (see Fig. S1E). When these are taken into account, *Ap*LdcA generally shows less turnover of non-cross-linked muropeptides than does *Ec*LdcA, likely indicating a reduced preference for these substrates relative to *Ec*LdcA.

Unlike the cell wall fragments that *Ec*LdcA might encounter during PGN recycling, mutanolysin-derived muropeptides contain cross-linked stem peptides that would normally be hydrolyzed by endopeptidases prior to being imported into the cytoplasm. Though *Ec*LdcA is not known to hydrolyze cross-linkages, we observed a decrease in the abundance of nearly all cross-linked compounds for both *Ec*LdcA and *Ap*LdcA-treated muropeptides, including both 4,3- and 3,3-cross-linked stem peptides (Fig. 3B). Among cross-linked compounds with two glycan substituents (one on each stem peptide), we observed greater turnover of substrates containing G-aM over GM glycans (2 G-aM > 1 G-aM and 1 GM > 2 GM) (Fig. 3B). Cross-linked compounds containing only one glycan (on either stem peptide), likely resulting from partial amidase activity, are generally less affected by either LdcA enzyme than di-substituted compounds, though G-aM-containing muropeptides are still preferred over those with GM (Fig. 3B). We found the same trends among some noncanonical cross-linked stem peptides (see Fig. S1G). In general, *Ap*LdcA treatment decreased the abundance of cross-linked muropeptides to a greater extent than *Ec*LdcA (Fig. 3B; see also Fig. S1F and G and Table S1).

Collectively, these results demonstrate that *Ap*LdcA can act as an l,d-endopeptidase on pentapeptide substrates and that both *Ec*LdcA and *Ap*LdcA are capable of hydrolyzing PGN cross-linkages, an activity previously not reported for *Ec*LdcA (47).

### LdcA is an l,d-endopeptidase toward both stem pentapeptides and cross-linkages.

**FIG 4:**
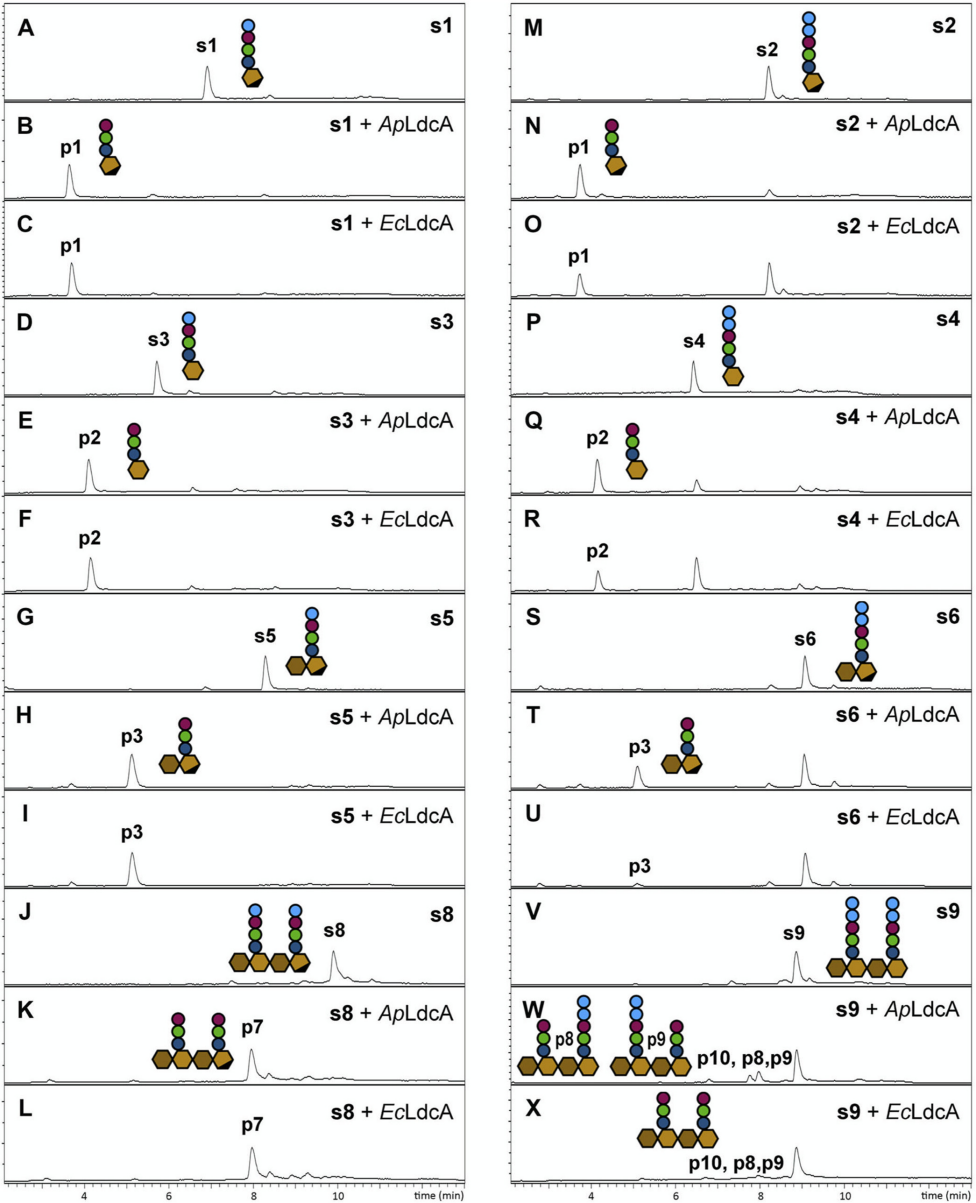
Single-substrate assays demonstrating the l,d-carboxypeptidase and l,d-endopeptidase activities of LdcA enzymes on stem tetrapeptides (A to L) and pentapeptides (M to X), respectively. LC-MS traces are shown for each reaction. Chemical structures of substrates (preceded by an “s”) are shown in Fig. S2. MS data are shown in Fig. S3 to S5.

**FIG 5:**
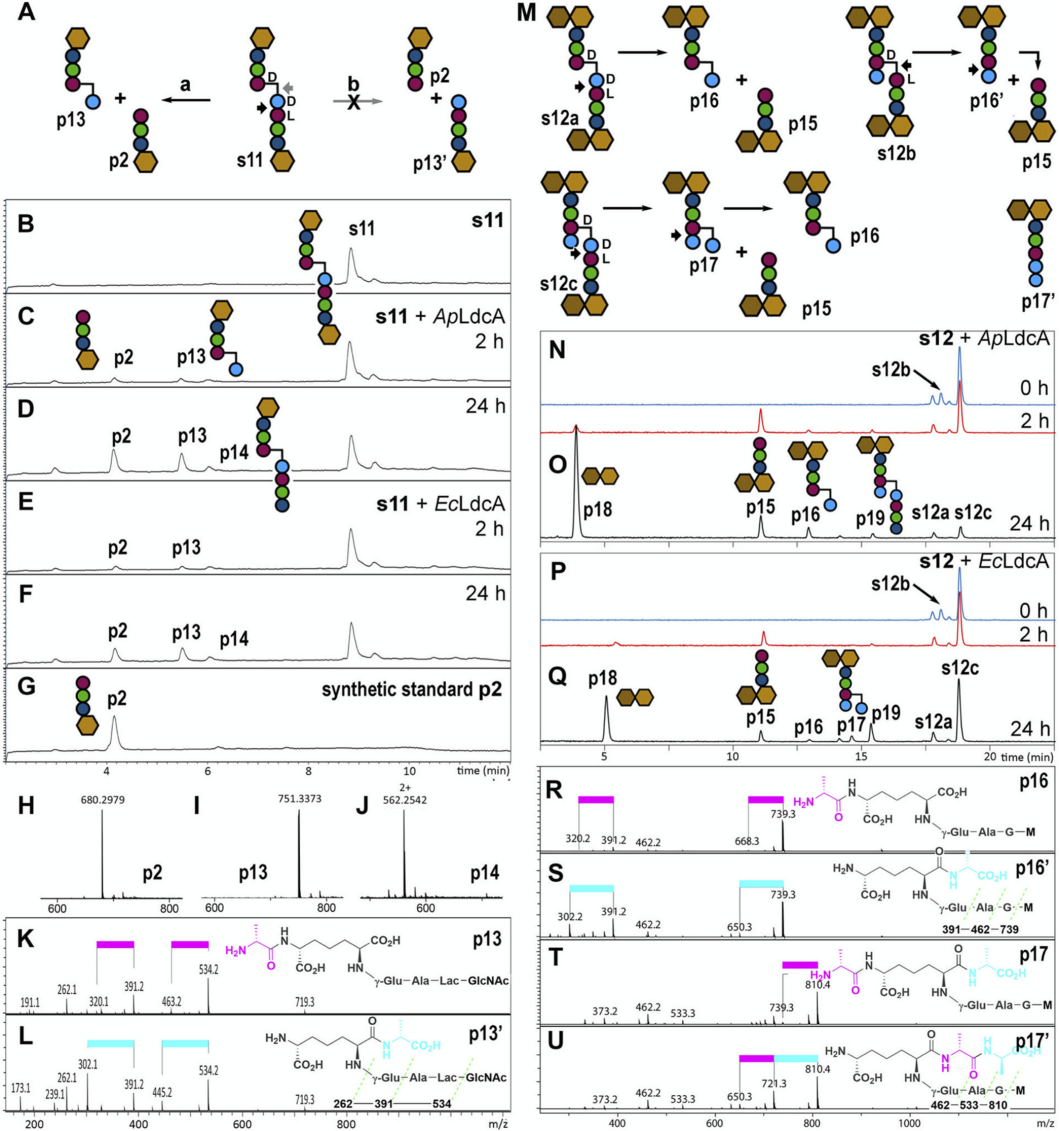
Single-substrate assays demonstrating the l,d-endopeptidase activity of LdcA enzymes on cross-linked muropeptides. (A) Two hydrolyzable bonds in s11 indicated with black and gray arrows correspond to routes “a” and “b,” respectively. (B to G) LC-MS traces of LdcA reactions with s11. (H to J) Mass spectra of s11 reaction products p2, p13, and p14. (K and L) Collision-induced dissociation (CID) MS/MS spectra of p13 and p13′ confirm hydrolysis of s11 by route “a.” The pink bar indicates the loss of Ala from the N terminus (71 Da), while the blue bar indicates the loss of Ala from the C terminus (89 Da). (M to Q) Reactions of LdcA with s12 (M) and the resulting substrate and product LC-MS traces (N to Q). (R to U) CID MS/MS spectra of detected route “a” products (p16 and p17) and potential route “b” products (p16′ and p17′). Further LC-MS/MS data are shown in Fig. S6.

We next sought to validate the results of our proteomic analysis of LdcA activity and to characterize the transformations for each LdcA enzyme with authentic synthetic muropeptide samples. The activities of the LdcA enzymes were assayed with each of 11 authentic PGN substrates produced by multistep chemical syntheses developed previously in our laboratory (Table 1; see also Fig. S2) (48, 49). Three different types of peptide were used to assess each activity observed above for LdcA—tetrapeptide for the l,d-carboxypeptidase activity, pentapeptide for the l,d-endopeptidase activity, and 4,3-cross-linked tetra-tripeptide for cross-linkage endopeptidase activity (Fig. 1). Substrate glycans also varied in length and composition (Table 1). Reactions were monitored by UPLC-MS, with products identified by an analysis of retention times, high-resolution mass measurements, and MS/MS spectra (Fig. 4 and 5; see also Fig. S3 to S6). Comparisons of reaction products to authentic synthetic standards were made whenever possible (Fig. 4 and 5; see also Fig. S3 to S6), and negative controls were included for most synthetic endopeptidase substrates (see Fig. S7 at https://figshare.com/articles/figure/Figure_S7_Negative_controls/16823347). In this section, we refer to specific substrates and products with a lowercase “s” and “p,” respectively, preceding a number referring to the structures shown in Fig. S2 to S6.

**TABLE 1:** Reactions of synthetic PGNs with *Ap*LdcA or *Ec*LdcA, given as the percentage of product formed following treatment of different peptide (column headers) plus glycan (rows) substrates with enzyme (columns)

Glycan^*a*^	Tetrapeptide (Ala-Glu*-m*Dap*=*Ala)		Pentapeptide (Ala-Glu*-m*Dap*=*Ala-Ala)		Tetra-tripeptide (4,3) (Ala-Glu-*m*Dap-Ala*=m*Dap-Glu-Ala)	
	*Ap*LdcA	*Ec*LdcA	*Ap*LdcA	*Ec*LdcA	*Ap*LdcA	*Ec*LdcA
mM	100	100	75	38	8/35 ∗4/6	7/22 ∗3/6
aM	100	100	84	41	18/66	13/29
G-mM	NA	NA	41/80 ∗0/12	18/56 ∗0/3	NA	NA
G-aM	100	100	43/89 ∗0/5	8/25 ∗0/0	NA	NA
GMG-mM	NA	NA	37/58 ∗0/12	14/51 ∗0/3	NA	NA
GMG-aM	100	100	NA	NA	NA	NA

a Product formation was determined after 2 h (single value) or at 2 and 24 h (values separated by “/”, respectively). Values preceded by an asterisk (∗) represent the percentage of *N*-acetyl muramyl l-Ala amidase product observed. For glycans, mM indicates that the MurNAc C-1 hydroxyl is replaced by β-OCH_3_, and aM indicates 1,6-anhydro-MurNAc. “NA” indicates where substrates were not available.

10.1128/mBio.02636-21.3 FIG S2 (A) Synthetic peptidoglycans (s1 to s11) and purified muropeptides (s12, a mixture of s12a, s12b, and s12c) used in this study. (B) Stem peptide chemical structures of l,d-carboxypeptidase and l,d-endopeptidase substrates and their corresponding cartoon representations. Each substrate shares the moiety within the dashed box. The white hexagon represents the variable glycan substitutions of substrates s1 to s12 (MurNAc, anhydro-MurNAc, GlcNAc-MurNAc, and GlcNAc-anhydro-MurNAc). Download FIG S2, TIF file, 0.7 MB. Copyright © 2021 Smith et al. 2021 Smith et al. https://creativecommons.org/licenses/by/4.0/ This content is distributed under the terms of the Creative Commons Attribution 4.0 International license.

10.1128/mBio.02636-21.4 FIG S3 (A, E, K, and O) Reactions of LdcA with monosaccharide compounds s1 to s4. LC-MS traces for substrates and products of reactions with s1 (B to D), s2 (F to I), s3 (L to N), and s4 (P to S). (J and T) Mass spectra of substrates, corresponding reaction products, and available synthetic standards. The *m*/*z* values shown for all MS peaks represent [M + H]^+^ ions. Download FIG S3, TIF file, 1.2 MB. Copyright © 2021 Smith et al. 2021 Smith et al. https://creativecommons.org/licenses/by/4.0/ This content is distributed under the terms of the Creative Commons Attribution 4.0 International license.

10.1128/mBio.02636-21.7 FIG S6 (A, M) Reactions of LdcA with cross-linked compounds s10 and s12, the latter representing a mixture of s12a, s12b, and s12c. The bond hydrolyzed by LdcA is shown in red. Two hydrolysable bonds in s10 indicated with green arrows correspond to reactions “a” and “b” of Fig. 5A, respectively. LC-MS traces for substrates and products of reactions with s10 (B to G) and s12 (N to T) are shown. (H to J, U) Mass spectra of substrate and reaction products. (K and L) CID MS/MS spectra of products p12 and s1. The pink bar indicates the loss of Ala at the N terminus (71 Da), while the blue bar indicates that of Ala at the C terminus (89 Da). The route “a” products were detected after treatment of s10 with LdcA (K). For each MS and MS/MS spectrum, the *m*/*z* values shown represent [M + H]^+^ ions unless labeled with “2+”, indicating an [M + 2H]^2+^ ion, and except for p18, which is an [M+Na]^+^ ion. Download FIG S6, TIF file, 1.4 MB. Copyright © 2021 Smith et al. 2021 Smith et al. https://creativecommons.org/licenses/by/4.0/ This content is distributed under the terms of the Creative Commons Attribution 4.0 International license.

All four tetrapeptide substrates, including three non-cross-linked muropeptides (s1, s3, and s5) and both stem peptides of a glycan-linked dimer (s8), were hydrolyzed completely to the corresponding tripeptide products by both *Ec*LdcA and *Ap*LdcA (Table 1, Fig. 4A to L; see also Fig. S3 to S5). Pentapeptide substrates (s2, s4, s6, s7, and s9), though not as rapidly consumed as tetrapeptide substrates, were also converted to tripeptide products by both *Ec*LdcA and *Ap*LdcA, with *Ap*LdcA demonstrating higher turnover ability than *Ec*LdcA with each substrate (Table 1 and Fig. 4M to X; see also Fig. S3 to S5). This disparity appears even more pronounced with disaccharide-pentapeptides (s6 and s7; Table 1; see also Fig. 4S to U) and glycan-linked pentapeptide dimers (s9; Table 1 and Fig. 4V to X; see also Fig. S5) than with monosaccharide pentapeptides (s2 and s4; Table 1 and Fig. 4M to R; see also Fig. S3). No products were detected in 24-h control reactions of pentapeptide substrates with either heat-inactivated enzymes or bovine serum albumin (BSA; see Fig. S7 at https://figshare.com/articles/figure/Figure_S7_Negative_controls/16823347), indicating that noncatalytic degradation does not occur under the conditions employed. It is possible, however, that trace amounts of unidentified peptidoglycan-degrading enzymes were copurified with the recombinant LdcA enzymes, such that products accumulated only after long incubation times. Tandem MS fragmentation patterns of tetra- and pentapeptide substrates and products are shown in Fig. S5.

10.1128/mBio.02636-21.5 FIG S4 (A, E, and R) Reactions of LdcA with disaccharide compounds s5 to s7. LC-MS traces for substrates and products of reactions with s5 (B to D), s6 (F to K), and s7 (S to X). (L to Q, Y) Mass spectra of substrates, reaction products, and synthetic standards. The *m*/*z* values shown for all MS peaks represent [M + H]^+^ ions. Download FIG S4, TIF file, 1.1 MB. Copyright © 2021 Smith et al. 2021 Smith et al. https://creativecommons.org/licenses/by/4.0/ This content is distributed under the terms of the Creative Commons Attribution 4.0 International license.

10.1128/mBio.02636-21.6 FIG S5 (A and E) Reactions of LdcA with dimeric compounds s8 and s9. LC-MS traces for substrates and products of reactions with s8 (B to D) and s9 (F to L). (M to S) Mass spectra of substrates and reaction products. The *m*/*z* values shown in panels M to S represent [M + 2H]^2+^ ions. The glycan GmM represents GlcNAc-MurNAc with the C-1 hydroxyl of MurNAc replaced by β-OCH_3_. (T) Structure elucidation of reaction substrates and products by CID MS/MS. The *m/z* values shown in T represent [M + H]^+^ ions. The red bar indicates the loss of CH_4_O (32 Da). Download FIG S5, TIF file, 1.4 MB. Copyright © 2021 Smith et al. 2021 Smith et al. https://creativecommons.org/licenses/by/4.0/ This content is distributed under the terms of the Creative Commons Attribution 4.0 International license.

We confirmed that LdcA exhibits cross-linkage endopeptidase activity and found that LdcA carries out a reaction that is not performed by any known endopeptidase. For 4,3-cross-linked tetra-tripeptide substrates (s10 and s11), there are two potential hydrolysable bonds (Fig. 5; see also Fig. S6), both of which yield tripeptide and tetrapeptide products. While the tripeptide product from either reaction is identical, the tetrapeptide products, though equivalent in mass, differ by the position of d-Ala on either the *m*Dap side chain (Fig. 5A; see also Fig. S6, route “a”) or the main chain (Fig. 5A; see also Fig. S6, route “b”). The two products are readily differentiated from their MS/MS fragmentation patterns, revealing that both *Ec*LdcA and *Ap*LdcA proceed through route “a” (Fig. 5K and L; see also Fig. S6), reinforcing the specificity of LdcA for cleavage of l,d-amide bonds. This specificity distinguishes LdcA endopeptidase activity from that of the d,d-endopeptidase activity of Pseudomonas aeruginosa penicillin-binding protein 4, which we previously showed turns over the same cross-linked substrate by route “b” (49). *Ap*LdcA was more active than *Ec*LdcA toward both cross-linked substrates (s10 and s11; Table 1 and Fig. 5B to G; see also Fig. S6), suggesting some differences in glycan preference. No substrate degradation or product formation was observed when 4,3-cross-linked substrates were treated with heat-inactivated enzymes or BSA (see Fig. S7 at https://figshare.com/articles/figure/Figure_S7_Negative_controls/16823347).

In our automated analysis, we observed a decreased abundance of 3,3-cross-linked tri-tripeptide compounds following LdcA treatment (Fig. 3B), but did not have synthetic 3,3-cross-linked substrates readily available to confirm this reaction. To address this remaining question, we purified a mixture of cross-linked substrates from mutanolysin-derived E. coli muropeptides (s12), containing 4,3-cross-linked tetra-tripeptide (s12a), 3,3-cross-linked tri-tetrapeptide (s12b), and 4,3-cross-linked tetra-tetrapeptide (s12c) in a ratio of ∼1:1:5, respectively. Figure 5M illustrates the reaction of LdcA with these three substrates. Reaction mixtures showed complete turnover of the 3,3-cross-linked s12b and partial turnover of 4,3-cross-linked compounds s12a and s12c, demonstrating that both *Ap*LdcA and *Ec*LdcA are more active against 3,3-cross-linkages than 4,3-cross-linkages (Fig. 5N to Q; see also Fig. S6). MepK is the only other enzyme known to display l,d-endopeptidase activity toward 3,3-cross-linkages (50). MepK is also capable of cleaving 4,3-cross-linkages, but via d,d-endopeptidase activity (Fig. 5M, route “b”) (50). Reaction of the 4,3-cross-linked substrates with LdcA was evident by the accumulation of route “a”-type tetrapeptide (p16) and pentapeptide (p17) products (Fig. 5N to Q). We confirmed that p16 and p17 are not the mass-equivalent route “b” products (p16′ and p17′) by comparison of their MS/MS fragmentation patterns (Fig. 5R to U). While p16 represents a reaction end product, p17 can be further converted to p16 by the l,d-carboxypeptidase activity of LdcA. Reactions with *Ap*LdcA showed accumulation of p16 only, while *Ec*LdcA treatment produced more p17 than p16 (Fig. 5N to Q; see also Fig. S6), suggesting that the conversion of p17 to p16 proceeds more slowly for *Ec*LdcA than *Ap*LdcA. This result may explain why the proportion of stem pentapeptides in mutanolysin-derived E. coli PGN decreased after treatment with *Ap*LdcA but increased for *Ec*LdcA (Fig. 3B).

Taken together, these results confirm the majority of our conclusions from the proteomic analysis of LdcA treatment on E. coli PGN (Fig. 3), demonstrating that: (i) *Ec*LdcA and *Ap*LdcA act as l,d-carboxypeptidases with un-cross-linked stem tetrapeptides and as l,d-endopeptidases with both 4,3- and 3,3-stem peptide cross-linkages, and (ii) *Ap*LdcA exhibits increased turnover of endopeptidase substrates relative to *Ec*LdcA (Table 1). The LdcA homolog from N. gonorrhoeae is also capable of hydrolyzing 3,3-cross-linkages *in vitro*; although the exact bond cleavage was not determined in that case, this finding suggests that the endopeptidase activity of this enzyme exhibits the same specificity for l,d-amide bonds that we observed for *Ec*LdcA and *Ap*LdcA (30). In addition, whereas our automated analysis successfully identified *Ap*LdcA as an l,d-endopeptidase with regard to un-cross-linked stem pentapeptides, single substrate analysis revealed that *Ec*LdcA, too, exhibits this activity (Table 1).

## DISCUSSION

Despite its role in PGN recycling, a cytoplasmic process involving soluble muropeptides, there is growing evidence that LdcA is an efficient starting point for the evolution of novel enzyme activities that modify the cell wall (29, 30). In aphids, the use of LdcA in their symbiotic association with *Buchnera* implies a derived ability of *Ap*LdcA to target symbiont cell walls. We found by biochemical characterization that both *Ec*LdcA and *Ap*LdcA exhibit l,d-carboxypeptidase and l,d-endopeptidase activities. The endopeptidase activities of *Ec*LdcA might be considered promiscuous, since the cytoplasmic *Ec*LdcA does not encounter cross-linked stem peptides in nature. In contrast, *Ap*LdcA is likely to encounter cross-linked *Buchnera* PGN because the enzyme is produced by the host, outside of symbiont cells. Whether *Ap*LdcA is transported into the *Buchnera* periplasm, where it directly remodels the cell wall, or is present in the bacteriocyte cytoplasm and acts on soluble muropeptides released during *Buchnera* cell wall remodeling is unknown. There is evidence of host protein transport to *Buchnera* for at least one host HTG, RlpA4 (51), although this enzyme contains a eukaryotic signal peptide that is absent from *Ap*LdcA (23). Because the endopeptidase activities of *Ec*LdcA are shared by *Ap*LdcA but are likely physiologically relevant in aphids, our results suggest an evolutionary link between the inherent enzyme promiscuity of *Ec*LdcA and the higher turnover of endopeptidase substrates by some extant LdcA enzymes, including *Ap*LdcA.

Catalytically promiscuous enzyme reactions vary greatly in magnitude among related proteins, sometimes approaching a level of efficiency similar to that of their primary functions (52). In addition, many models of protein evolution emphasize enzymatic tradeoffs between promiscuous and primary activities (1). Nonetheless, *Ap*LdcA and some other multifunctional LdcA enzymes retain a high level of l,d-carboxypeptidase activity (Fig. 3) (29–31). Thus, enzyme function is not easily distinguished from promiscuity without a demonstrable role in biology. We provide evidence that each catalytic activity of *Ap*LdcA plays a role in the aphid-*Buchnera* symbiosis, implying selection for multifunctionality. Some of the muropeptides identified from aphid homogenate are likely the products of *Buchnera* cell wall digestion by *Ap*LdcA, implying its functionality *in vivo* (Fig. 2). Specifically, tripeptide monomers can only be formed from tetrapeptides or by the cleavage of stem peptide cross-linkages via l,d-carboxypeptidase or endopeptidase activities, respectively, both of which can be catalyzed by *Ap*LdcA and are not encoded elsewhere in the *Buchnera* or host genomes. Though additional enzyme functions cannot be ruled out for the other PGN-modifying enzymes that are retained in the *Buchnera* genome, our data indicate that these roles can be fulfilled by the catalytic functions of *Ap*LdcA.

Several LdcA enzymes from bacteria have now been biochemically characterized and, in conjunction with our own results, some patterns emerge that hint at the evolutionary origin of enhanced endopeptidase functions within the LdcA family. The *A. pisum ldcA* gene is thought to have been acquired by an ancient aphid ancestor from *Wolbachia*-like bacteria (22), which are frequently associated with arthropod hosts as intracellular pathogens or facultative symbionts (53). Given that all other LdcA enzymes known to target cell wall and display endopeptidase activity are derived from intracellular bacteria, we hypothesize that the *ldcA* gene originally acquired by ancestral aphids already exhibited these abilities and provided an immediate advantage to the insect host, possibly enabling aphids to establish the high degree of control over *Buchnera* that exists today. An analysis of crosswise promiscuity within the LdcA family that includes both extant and ancestrally reconstructed enzymes of free-living, intracellular, and aphid origins could be used to test this idea.

Besides *Ap*LdcA, aphids harbor six other HTGs with putative roles in PGN remodeling and symbiosis: *amiD*, encoding an amidase, and *rlpA1* to *rlpA5*, all encoding lytic transglycosylases (Fig. 1). Together with *Ap*LdcA, they possess each of the necessary enzyme functions required for PGN remodeling in free-living bacteria (54). Based on their high levels of bacteriocyte-specific expression (23), their importance for both aphid and symbiont growth (24), and their correlation with high symbiont abundance among distinct aphid genotypes (25), these genes appear to contribute to *Buchnera* proliferation. HTGs could be involved in releasing cell wall fragments that mediate host-microbe interactions (55) or in degrading those fragments as a means for hosts to curb their own immune response to indigenous microbiota (56, 57). However, because aphids lack PGRPs (58), it seems unlikely that *Buchnera* cell wall fragments affect aphid hosts at all. Though it is possible that some other aphid signaling pathway has been coopted for recognition of Gram-negative PGN, we propose an alternative hypothesis in which host control of PGN metabolism enables aphids to regulate symbiont PGN metabolism and regulate their growth and/or cell division.

Multifunctionality is apparently common among individual PGN hydrolase domains (29, 30, 59–61), suggesting that multifunctionality in the other aphid HTGs or in *Buchnera*’s own PGN remodeling enzymes may exist. For example, *Buchnera* contains AmiB and the typically nonenzymatic NlpD. In E. coli, NlpD is the designated activator of AmiC, while EnvC, missing in *Buchnera*, activates AmiA and AmiB (62). In Waddlia chondrophila and Chlamydia pneumoniae, NlpD acts as a bifunctional d,d-carboxypeptidase and d,d-endopeptidase, independent of any amidase (59, 60). In addition, E. coli PBP1B exhibits d,d-carboxypeptidase activity under acidic conditions and in the presence of its activator, LpoB (61)—both of these proteins are encoded by *Buchnera.* Thus, in addition to *Ap*LdcA, either *Buchnera* NlpD or PBP1B could contribute to the production of trimmed stem peptides among muropeptides from aphid homogenate (Fig. 2). In another example of multifunctionality, *Buchnera* lacks Alr and DadX, each capable of producing d-Ala for lipid II biosynthesis via alanine-racemase activity, but contains GlyA, to which the alanine-racemase activity of C. pneumoniae has been attributed (63). Our results support the idea that aphid PGN hydrolases are involved in *Buchnera* PGN metabolism, but further interrogation of these pathways is required to understand how host enzymes contribute. To add another layer of complexity, *Buchnera* symbionts of different aphid species vary greatly in PGN gene repertoire (64, 65), which could translate to substantial differences in cell wall and/or enzyme chemistry depending on their metabolic needs.

In conclusion, aphids encode each of the three enzyme functions required for PGN remodeling. While both *Ec*LdcA and *Ap*LdcA behave as endopeptidases, *Ap*LdcA exhibits enhanced endopeptidase activity toward pentapeptides and cross-linked dimers, revealing adaptations in this enzyme specific to the aphid-*Buchnera* system. Host acquisition and retention of these HTGs throughout aphid evolution reflect their importance in symbiosis. Potentially, their acquisition by aphid ancestors was instrumental in establishing control over symbionts that has since evolved further in extant aphids.

## MATERIALS AND METHODS

### Purification of PGN.

*Buchnera* muropeptides were purified from homogenates of 7-day-old fourth-instar *A. pisum* nymphs by successive filtration. The filtrate was subjected to HPLC, and the collected fractions were subjected to proteomic analysis. Whole E. coli DH5α murein sacculus was purified following the methods of Desmarais et al. (66). More details are provided in the supplemental Materials and Methods (see Text S1).

10.1128/mBio.02636-21.1 TEXT S1 Supplemental results: additional single-substrate enzyme assay results reporting the minor accumulation of amidase products in LdcA enzyme reactions. Supplemental methods: detailed descriptions of the methods used in this work. Download Text S1, DOCX file, 0.08 MB. Copyright © 2021 Smith et al. 2021 Smith et al. https://creativecommons.org/licenses/by/4.0/ This content is distributed under the terms of the Creative Commons Attribution 4.0 International license.

### Protein production and purification.

The *A. pisum* and E. coli *ldcA* genes were amplified by PCR using primers in Table S2, then cloned into the pET-28b plasmid. E. coli Rosetta (DE3) was transformed with plasmid and induced with isopropyl β-d-1-thioglactopyranoside (IPTG). Proteins were purified from cell pellets by (i) lysis with HEWL and high-pressure homogenization, (ii) binding to Ni-NTA IMAC resin, and (iii) elution with imidazole.

10.1128/mBio.02636-21.9 TABLE S2 Primers used to construct plasmids for recombinant protein expression. *T_m_* values were calculated for the specific DNA polymerase used for each primer set. Sequences in boldface denote restriction-enzyme recognition sites, while underlined sequences highlight the target sequence used to calculate the *T_m_*. Download Table S2, DOCX file, 0.06 MB. Copyright © 2021 Smith et al. 2021 Smith et al. https://creativecommons.org/licenses/by/4.0/ This content is distributed under the terms of the Creative Commons Attribution 4.0 International license.

### Proteomics-based muropeptide analysis.

E. coli sacculus was digested with mutanolysin (Sigma-Aldrich) and the resulting muropeptides treated with sodium borohydride and desalted using HPLC. Muropeptides were incubated with LdcA enzyme and then desalted again by HPLC. Treated samples were subjected to nano LC-MS/MS analysis following the methods of Bern et al. (37). Individual PGN compounds were identified and quantified from MS and MS/MS spectra using Byonic and Byologic software, respectively (Protein Metrics). All MS data and associated Byonic and Byologic files are available for download via the mass spectrometry database MassIVE (MSV000087634). Data transformation, statistical comparison, and plotting were accomplished using custom R scripts, available along with raw and transformed data at GitHub (https://github.com/smit4227/ApLdcA_proteomics).

### Single-substrate enzyme assays.

Synthetic PGN substrates (s1 to s11) used in this study were synthesized using previously reported methods (48, 49). The muropeptide mixture s12 was purified from mutanolysin-derived E. coli muropeptides by HPLC. Reactions of LdcA enzymes with synthetic PGNs were stopped at different time points (2, 8, and 24 h) and analyzed by UPLC-MS. Reactions of LdcA enzymes with s12 were carried out under the same conditions, except that reaction mixtures were reduced with sodium borohydride after treatment.
