# Supplementary material for: Horizontal-Acquisition of a Promiscuous Peptidoglycan-Recycling Enzyme Enables Aphids To Influence Symbiont Cell Wall Metabolism
Source: mBio. 2021 Dec 21;12(6):e02636-21. doi: 10.1128/mBio.02636-21 (PMC8689515; doi:10.1128/mBio.02636-21)
Supplement: TABLE S1 [file mbio.02636-21-st001.docx]

| **Compound** | | | | | **Average percent total PGN** | | | |
| --- | --- | --- | --- | --- | --- | --- | --- | --- |
| **#** | **stem peptide** | **crosslinkage acceptor** | **GM** | **GaM** | ***E. coli*^a,b^** | ***Buchnera*^a^** | ***Ec*LdcA^b^** | ***Ap*LdcA^b^** |
| 1 | tri | NA^c^ | 1 | 0 | 1.22E-01 | 2.11E+00 | 1.72E+00 | 2.15E+00 |
| 2 | tri | NA^c^ | 0 | 1 | 6.93E-03 | 3.78E-02 | 6.65E-02 | 1.46E-01 |
| 3 | tetra | NA^c^ | 1 | 0 | 1.45E+01 | 5.25E+00 | 8.70E-02 | 7.08E-01 |
| 4 | tetra | NA^c^ | 0 | 1 | 2.54E+00 | 5.64E+00 | 9.56E-03 | 1.79E-01 |
| 5 | penta | NA^c^ | 1 | 0 | 1.16E-01 | 4.60E+00 | 1.34E+00 | 4.75E-02 |
| 6 | penta | NA^c^ | 0 | 1 | 1.94E-03 | 1.95E-01 | 7.97E-03 | 2.22E-04 |
| 7 | tri | tri | 2 | 0 | 7.92E-01 | 1.28E+01 | 1.12E-01 | 9.04E-02 |
| 8 | tri | tri | 1 | 1 | 4.78E-01 | 1.45E-01 | 1.40E-02 | 2.96E-02 |
| 9 | tri | tri | 0 | 2 | 1.65E-02 | 1.09E-04 | 2.60E-05 | 2.32E-05 |
| 10 | tri | tri | 1 | 0 | 3.06E-02 | 0.00E+00 | 6.91E-03 | 3.59E-03 |
| 11 | tetra / tri | tri / tetra | 2 | 0 | 9.93E+00 | 4.56E+00 | 4.52E+00 | 3.61E+00 |
| 12 | tetra / tri | tri / tetra | 1 | 1 | 3.12E+00 | 6.51E-01 | 4.83E-01 | 2.04E-01 |
| 13 | tetra / tri | tri / tetra | 0 | 2 | 5.43E-02 | 1.79E-02 | 3.62E-03 | 4.08E-04 |
| 14 | tetra / tri | tri / tetra | 1 | 0 | 4.70E-01 | 0.00E+00 | 2.48E-01 | 1.97E-01 |
| 15 | tetra / tri | tri / tetra | 0 | 1 | 3.37E-02 | 1.31E-01 | 7.01E-03 | 1.14E-03 |
| 16 | tetra / tri | tetra / penta | 2 | 0 | 2.56E+01 | 3.86E+01 | 4.33E+01 | 4.62E+01 |
| 17 | tetra / tri | tetra / penta | 1 | 1 | 6.63E+00 | 2.51E+00 | 2.62E+00 | 4.04E-01 |
| 18 | tetra / tri | tetra / penta | 0 | 2 | 5.92E-02 | 5.11E-02 | 6.39E-03 | 3.92E-04 |
| 19 | tetra / tri | tetra / penta | 1 | 0 | 2.00E+00 | 6.97E+00 | 1.93E+00 | 1.39E+00 |
| 20 | tetra / tri | tetra / penta | 0 | 1 | 9.21E-02 | 3.11E-01 | 6.35E-02 | 1.10E-02 |
| 21 | tetra | penta | 2 | 0 | 2.32E-01 | 0.00E+00 | 3.81E-01 | 3.21E-01 |
| 22 | tetra | penta | 1 | 1 | 7.14E-02 | 0.00E+00 | 4.05E-02 | 1.14E-02 |
| 23 | tetra | penta | 0 | 1 | 6.20E-03 | 3.15E-01 | 1.09E-02 | 1.11E-02 |
| 24 | tetra | penta | 0 | 0 | 2.41E-07 | 5.18E-01 | 4.74E-06 | 5.64E-06 |
| 25 | tri | tetra-tetra | 2 | 0 | 0.00E+00 | 1.82E-03 | 0.00E+00 | 0.00E+00 |
| 26 | tetra / tri | tetra-tetra/penta | 2 | 0 | 2.13E-02 | 0.00E+00 | 2.34E-02 | 4.04E-02 |
| 27 | tetra / tri | tetra-tetra/penta | 1 | 0 | 2.09E-03 | 0.00E+00 | 9.45E-04 | 1.03E-03 |
| 28 | tetra / tri | tetra-tetra/penta | 0 | 0 | 3.02E-06 | 2.18E-01 | 1.45E-03 | 1.66E-03 |
| 29 | tetra | tetra-penta | 1 | 0 | 5.28E-03 | 3.15E-01 | 2.05E-02 | 3.50E-02 |
| 30 | tetra | tetra-penta | 0 | 0 | 4.50E-06 | 5.18E-01 | 4.50E-06 | 3.03E-05 |
| 31 | tetra (Gly^4^) | NA^c^ | 1 | 0 | 8.71E-02 | 0.00E+00 | 3.34E-05 | 5.16E-04 |
| 32 | tetra (Gly^4^) | NA^c^ | 0 | 1 | 3.33E-02 | 4.37E-02 | 1.02E-04 | 1.50E-04 |
| 33 | tetra (Ser^4^) | NA^c^ | 1 | 0 | 1.36E-02 | 0.00E+00 | 2.21E-04 | 1.84E-04 |
| 34 | tetra (Val^4^) | NA^c^ | 1 | 0 | 3.61E-02 | 0.00E+00 | 1.89E-04 | 6.15E-03 |
| 35 | tetra (Cys^4^) | NA^c^ | 1 | 0 | 5.44E-02 | 0.00E+00 | 4.06E-04 | 9.65E-03 |
| 36 | tetra (Ile/Leu^4^) | NA^c^ | 1 | 0 | 2.50E-02 | 0.00E+00 | 2.43E-05 | 6.27E-03 |
| 37 | tetra (Asn^4^) /  penta (Gly^4^-Gly^5^) | NA^c^ | 1 | 0 | 1.95E-02 | 0.00E+00 | 3.85E-04 | 9.66E-04 |
| 38 | tetra (Asn^4^) /  penta (Gly^4^-Gly^5^) | NA^c^ | 0 | 1 | 1.12E-02 | 1.39E-01 | 1.71E-05 | 8.82E-05 |
| 39 | tetra (Gln^4^) /  penta (Ala^4^-Gly^5^) | NA^c^ | 1 | 0 | 3.01E-02 | 2.53E-01 | 6.96E-03 | 3.55E-04 |
| 40 | tetra (Lys^4^) | NA^c^ | 1 | 0 | 5.36E-02 | 8.88E-02 | 2.30E-04 | 7.89E-02 |
| 41 | tetra (Lys^4^) | NA^c^ | 0 | 1 | 9.35E-02 | 3.10E-02 | 3.05E-04 | 1.33E-01 |
| 42 | tetra (Glu^4^) | NA^c^ | 1 | 0 | 1.31E-02 | 0.00E+00 | 2.10E-03 | 1.06E-03 |
| 43 | tetra (Met^4^) | NA^c^ | 1 | 0 | 3.01E-02 | 0.00E+00 | 1.90E-05 | 1.88E-04 |
| 44 | tetra (His^4^) | NA^c^ | 1 | 0 | 4.60E-03 | 0.00E+00 | 5.80E-06 | 2.14E-04 |
| 45 | tetra (His^4^) | NA^c^ | 0 | 1 | 3.23E-02 | 2.65E-02 | 1.61E-04 | 4.49E-03 |
| 46 | tetra (Phe^4^) | NA^c^ | 1 | 0 | 6.24E-01 | 3.72E-06 | 1.52E-03 | 7.13E-02 |
| 47 | tetra (Phe^4^) | NA^c^ | 0 | 1 | 2.25E-02 | 1.01E-02 | 4.49E-06 | 5.54E-04 |
| 48 | tetra (Tyr^4^) | NA^c^ | 1 | 0 | 5.71E-02 | 0.00E+00 | 1.98E-04 | 3.35E-03 |
| 49 | tetra (Trp^4^) | NA^c^ | 1 | 0 | 2.59E-02 | 2.14E-03 | 2.04E-05 | 1.14E-02 |
| 50 | penta (Lys^4^-Arg^5^) | NA^c^ | 1 | 0 | 1.21E-01 | 4.61E+00 | 1.18E-02 | 2.21E-01 |
| 51 | penta (Lys^4^-Arg^5^) | NA^c^ | 0 | 1 | 1.25E-01 | 5.48E-02 | 1.18E-03 | 3.07E-01 |
| 52 | tri | tetra (Gly^4^) | 2 | 0 | 2.82E-01 | 0.00E+00 | 2.01E-03 | 2.54E-03 |
| 53 | tri | tetra (Gly^4^) | 1 | 1 | 1.05E-01 | 0.00E+00 | 2.27E-04 | 3.49E-04 |
| 54 | tri | tetra (Gly^4^) | 1 | 0 | 4.14E-03 | 0.00E+00 | 2.43E-03 | 1.15E-03 |
| 55 | tetra / tri / tri | tetra (Gly^4^)/  tetra (Gln^4^)/  penta (Ala^4^-Gly^5^) | 2 | 0 | 1.86E+00 | 2.63E-01 | 2.15E+00 | 1.82E+00 |
| 56 | tetra / tri / tri | tetra (Gly^4^)/  tetra (Gln^4^)/  penta (Ala^4^-Gly^5^) | 1 | 1 | 3.10E-01 | 5.31E-03 | 1.38E-01 | 4.68E-02 |
| 57 | tetra / tri / tri | tetra (Gly^4^)/  tetra (Gln^4^)/  penta (Ala^4^-Gly^5^) | 0 | 2 | 3.35E-03 | 0.00E+00 | 4.65E-04 | 2.49E-05 |
| 58 | tetra / tri / tri | tetra (Gly^4^)/  tetra (Gln^4^)/  penta (Ala^4^-Gly^5^) | 1 | 0 | 4.42E-02 | 0.00E+00 | 4.30E-02 | 3.56E-02 |
| 59 | tri | tetra (Val^4^) | 2 | 0 | 4.50E-02 | 0.00E+00 | 2.89E-02 | 1.85E-02 |
| 60 | tri | tetra (Val^4^) | 1 | 1 | 6.46E-03 | 0.00E+00 | 1.25E-03 | 3.89E-04 |
| 61 | tri | tetra (Asn^4^)/  penta (Gly^4^-Gly^5^) | 2 | 0 | 1.43E-02 | 0.00E+00 | 9.17E-04 | 1.00E-03 |
| 62 | tri | tetra (Asn^4^)/  penta (Gly^4^-Gly^5^) | 1 | 1 | 1.82E-01 | 0.00E+00 | 1.07E-01 | 8.05E-02 |
| 63 | tetra | tetra (Asn^4^)/  penta (Gly^4^-Gly^5^) | 2 | 0 | 2.24E-01 | 0.00E+00 | 1.80E-01 | 1.66E-01 |
| 64 | tetra | tetra (Asn^4^)/  penta (Gly^4^-Gly^5^) | 1 | 1 | 4.07E-01 | 0.00E+00 | 9.54E-01 | 1.13E+00 |
| 65 | tetra | tetra (Asn^4^)/  penta (Gly^4^-Gly^5^) | 1 | 0 | 3.04E-03 | 0.00E+00 | 3.43E-03 | 3.95E-03 |
| 66 | tri | tetra (Asp^4^) | 1 | 1 | 2.16E-01 | 0.00E+00 | 1.75E-01 | 1.47E-01 |
| 67 | tri | tetra (Asp^4^) | 0 | 2 | 9.91E-02 | 2.04E-02 | 2.12E-02 | 7.94E-03 |
| 68 | tri | tetra (Asp^4^) | 0 | 1 | 2.20E-03 | 7.43E-04 | 1.16E-03 | 1.29E-03 |
| 69 | tetra | tetra (Asp^4^) | 1 | 1 | 3.28E-01 | 0.00E+00 | 1.19E+00 | 1.62E+00 |
| 70 | tetra | tetra (Asp^4^) | 0 | 2 | 2.28E-01 | 1.65E-01 | 1.40E-01 | 2.43E-02 |
| 71 | tetra | tetra (Asp^4^) | 0 | 1 | 2.20E-02 | 2.71E-03 | 3.48E-02 | 2.50E-02 |
| 72 | tetra | tetra (Gln^4^)/  penta (Ala^4^-Gly^5^) | 2 | 0 | 4.56E-01 | 0.00E+00 | 4.89E-01 | 4.21E-01 |
| 73 | tetra | tetra (Gln^4^)/  penta (Ala^4^-Gly^5^) | 1 | 1 | 1.26E-01 | 0.00E+00 | 4.62E-02 | 1.16E-02 |
| 74 | tetra | tetra (Gln^4^)/  penta (Ala^4^-Gly^5^) | 1 | 0 | 1.08E-02 | 0.00E+00 | 1.01E-02 | 9.41E-03 |
| 75 | tri | tetra (Glu^4^) | 1 | 0 | 9.03E-03 | 0.00E+00 | 5.60E-05 | 1.80E-04 |
| 76 | tetra | tetra (Glu^4^) | 1 | 0 | 9.27E-02 | 0.00E+00 | 5.21E-02 | 3.57E-02 |
| 77 | tri | tetra (Phe^4^) | 2 | 0 | 2.22E-02 | 2.44E-03 | 2.92E-04 | 3.34E-04 |
| 78 | tri | tetra (Phe^4^) | 1 | 1 | 4.27E-01 | 0.00E+00 | 2.49E-01 | 2.64E-01 |
| 79 | tetra | tetra (Phe^4^) | 2 | 0 | 1.63E-01 | 1.12E-02 | 3.00E-02 | 1.83E-02 |
| 80 | tetra | tetra (Phe^4^) | 1 | 1 | 7.33E-03 | 0.00E+00 | 1.86E-04 | 1.23E-04 |
| 81 | tetra | tetra (Phe^4^) | 1 | 0 | 2.56E-03 | 0.00E+00 | 2.34E-03 | 2.12E-03 |
| 82 | tri | penta (Lys^4^-Arg^5^) | 2 | 0 | 4.98E+00 | 7.71E-01 | 2.11E-02 | 9.28E-02 |
| 83 | tri | penta (Lys^4^-Arg^5^) | 1 | 1 | 1.17E+00 | 0.00E+00 | 3.80E-03 | 1.84E-02 |
| 84 | tri | penta (Lys^4^-Arg^5^) | 0 | 2 | 3.05E-02 | 1.37E-02 | 6.38E-05 | 2.58E-04 |
| 85 | tri | penta (Lys^4^-Arg^5^) | 1 | 0 | 2.25E-02 | 0.00E+00 | 6.83E-03 | 3.51E-03 |
| 86 | tri | penta (Lys^4^-Arg^5^) | 0 | 1 | 8.53E-03 | 3.05E-02 | 5.38E-04 | 5.39E-04 |
| 87 | tetra | penta (Lys^4^-Arg^5^) | 2 | 0 | 1.57E+01 | 3.49E+00 | 3.10E+01 | 3.37E+01 |
| 88 | tetra | penta (Lys^4^-Arg^5^) | 1 | 1 | 3.84E+00 | 4.04E-01 | 5.67E+00 | 3.52E+00 |
| 89 | tetra | penta (Lys^4^-Arg^5^) | 0 | 2 | 5.17E-02 | 3.08E-03 | 6.33E-02 | 1.67E-02 |
| 90 | tetra | penta (Lys^4^-Arg^5^) | 1 | 0 | 9.13E-02 | 2.39E-01 | 8.15E-02 | 7.87E-02 |
| 91 | tetra | penta (Lys^4^-Arg^5^) | 0 | 1 | 1.06E-02 | 5.87E-02 | 7.18E-03 | 4.65E-03 |

^a^Data shown in Figures 2, S1A-B; ^b^Data shown in Figures 3, S1E-G; ^c^NA: not applicable—no acceptor stem peptide for non-crosslinked stems; ^4,5^Denotes the residue replacing d-Ala in fourth and fifth position(s) of stem peptide, respectively
