# Supplementary material for: Horizontal-Acquisition of a Promiscuous Peptidoglycan-Recycling Enzyme Enables Aphids To Influence Symbiont Cell Wall Metabolism
Source: mBio. 2021 Dec 21;12(6):e02636-21. doi: 10.1128/mBio.02636-21 (PMC8689515; doi:10.1128/mBio.02636-21)
Supplement: TEXT S1 [file mbio.02636-21-s0001.docx]

Supplementary Text for

**Horizontal-acquisition of a promiscuous peptidoglycan-recycling enzyme enables host control of symbiont cell wall remodeling in aphids**

Thomas E. Smith^a#^, Mijoon Lee^b,c^, Maria D. Person^d^, Dusan Hesek^b^, Shahriar Mobashery^b^, Nancy A. Moran^a^

E-mail: smit4227@gmail.com

**This PDF file includes:**

Supplementary Results

Supplementary Materials and Methods

SI References

**Supplementary Results**

**Detection of amidase activity in LdcA reactions*.*** We obserevd a low-level of amidase activity in LdcA reactions with substrates containing pentapeptide and crosslinked stems, especially in reactions with longer incubation times (24h) (Table 1). Amidase products included glycans lacking stem peptides (**p4**, **p6**, **p11** in Figures S4-S5 and **p18** and Figure 5O, Q) and glycanless stem peptides (**p14** in Figure 5D, F, J and **p19** in Figure 5O, Q). Products accumulated in similar abundances in reactions with either *Ec*LdcA or *Ap*LdcA. Amidase activity has not previously been reported for LdcA enzymes, and we include it here for the sake of transparency—additional experiments are necessary to rule out any influence from protein contaminants. However, the selectivity of LdcA for l,d-amide bonds suggests that the amide bond linking stem peptide and MurNAc (between l-Ala and d-lactyl) could conceivably be cleaved by LdcA.

**Supplementary Materials and Methods**

**Aphid maintenance.** *A. pisum* strain LSR1, reared on *Vicia faba* at 20˚C with a 14L/10D daily light cycle, were used for all experiments. Seven day-old 4^th^ instar aphid nymphs born within a 24 h period of each other were used for each experiment.

**Purification of PGN.** *Buchnera* muropeptides were purified aphid homogenate. Aphids were surface-sterilized inside a mesh tea ball by submerging in bleach for 1 min (400 ml; 0.5%) and rinsing twice with deionized water (400 ml). Aphids were then homogenized by mortar and pestle in buffer B (40 ml; 25 mM KCl, 35 mM Tris, 10 mM MgCl_2_, 25 mM EDTA, pH 7.5). At this point, HEWL could be added (0.4 mg/ml) and the homogenate incubated for 1 hour at 4˚C on a rocking platform. Homogenate was then sonicated on ice for 5 min using a 1/8 inch probe (30% amplitude, 10 s pulse, 15 s pause), and centrifuged for 5 min at 4000 rpm, 4˚C. The supernatant was successively filtered at room temperature through a 5 µm nylon mesh filter (EMD Millipore), 1 µm mixed cellulose ester filter (Advantec), and 0.2 µm PES membrane (EZFlow), before a final filtration via a 10 kDa MWCO centrifugal filter (Amicon), spinning overnight at 7800 rpm, 4˚C. The filtrate was frozen at -80˚C and lyophilized. The resulting material was suspended in deionized water (3 ml) and frozen prior to analysis. Muropeptides were purified from 25 mg of concentrated filtrate by HPLC using a Beckman System Gold 126 solvent module with 168 PDA detector, 32 Karat Software (Beckman-Coulter; version 8.0), and a Waters Nova-Pak C18 column (3.9 x 150 mm, 4 µm) at a flow rate of 1 ml/min with the following buffers (A = 0.1% formic acid, B = 100% acetonitrile) and method: hold at 1% B for 3 min, increase to 50% B over 2 min, hold at 50% B for 5 min, increase to 95% B over 1 min, decrease to 1% B over 4 min, hold at 1% B for 5 min. Three fractions were collected: prior to 3.5 min, following 6 min, and between 3.5 and 6 min, with the final fraction containing muropeptides. Each fractions was dried to completion by speedvac and frozen at -20˚C.

Whole *E. coli* murein sacculi were purified following the methods of Desmarais *et al.* (1). Briefly, LB media (10 ml) was inoculated with a single colony of DH5α *E. coli* and shaken at 37˚C, 220 rpm overnight. The overnight culture was diluted in fresh LB culture (1 L), which was shaken at 37˚C, 220 rpm until an OD_600_ of 0.7 was reached. The culture was centrifuged for 30 min at 4000 rpm, 25˚C. The cell pellet was resuspended in PBS buffer (3 ml) and added dropwise to a suspension of boiling SDS (6 ml; 6%) and boiled for 3 hours. Cell lysate was cooled to room temperature overnight, then centrifuged at 200,000 *g*, 25˚C for 20 min. The pellet was washed with deionized water (9 ml) and centrifuged an additional 3-4 times to remove excess detergent, then suspended in Tris buffer (900 µl; 10 mM, 0.06% NaCl, pH 7.5). Cell-wall proteins were digested with activated pronase (100 µl; 1 mg/ml, EMD Millipore) for two hours at 60˚C. Pronase was inactivated by boiling with SDS (200 µl; 6%) at 100˚C for 30 min. Protein-free pellets were centrifuged and washed with deionized water 3-4 times as described above. Pellets were resuspended in deionized water (1 ml), frozen at -80˚C, and lyophilized overnight.

**Construction of protein expression vectors.** The *A. pisum ldcA* gene (100168405) was amplified by PCR from cDNA prepared from whole, 4^th^ instar LSR1 aphids and cloned into the pET-28b bacterial expression vector as N-terminally His-tagged coding sequences. Total RNA was extracted from several aphid nymphs using the RNeasy Plus Mini kit following the animal tissues protocol using a needle and syringe for homogenization (Qiagen), and cDNA was synthesized from 100 ng of total RNA using the Verso cDNA Synthesis Kit (ThermoFisher). DNA concentrations were determined from UV absorbance (λ = 260 nm) measured on a NanoDrop Lite spectrophotometer (Thermo). Amplification of *ldcA* was performed with Phusion® DNA polymerase (NEB), primers 1 and 2 (Table S2), 1 ng of cDNA template per 10 µl reaction, and the following thermocycler program: an initial denaturation (98˚C, 1 min) followed by 35 cycles of denaturation (98˚C, 15 s), annealing (61-66˚C, 15 s), and elongation (72˚C, 35 s) and a final elongation step (72˚C, 5 min).

The *E. coli ldcA* gene was cloned into the same multiple-cloning site of pET-28b as the aphid homolog. Genomic DNA was purified from *E. coli* DH5α using the DNeasy Blood and Tissue kit following the protocol for Gram-negative bacteria (Qiagen). PCR amplification of *E. coli ldcA* was carried out using the same thermocycler program described above with Phusion DNA polymerase (NEB), 1:200 dilution of gDNA template per reaction, and primers 3 and 4 (Table S2).

For both constructs, amplified DNA was purified using the QIAquick PCR Purification kit (Qiagen). Amplified DNA and pET-28b vector were digested with NdeI-HF and XhoI restriction enzymes (NEB) and the cut vector dephorphorylated using Antarctic Dephosphorylase (NEB). Digested inserts were ligated into vector by inclubating with T4 DNA ligase (NEB) at 4˚C overnight. Ligation reactions were transformed into competent DH5α cells, plated on LB agar plates with kanamycin (50 µg/ml), and grown at 37˚C overnight. Colonies were screened for the expected insert size by colony PCR using Taq Polymerase (ThermoFisher) with primers 5 and 6 (Table S2) and the following thermocycler program: an initial denaturation (94˚C, 3 min) followed by 30 cycles of denaturation (94˚C, 30s), annealing (46˚C, 30s), and elongation (72˚C, 80s), and a final elongation step (72˚C, 7 min). Positive colonies were grown in LB liquid culture (10 ml) plus kanamycin (50 µg/ml) at 37˚C, 220 rpm overnight and plasmids purified using the QIAprep Spin Miniprep kit (Qiagen). Purified plasmids were verified by Sanger sequencing using primers 5 and 6 (Table S2).

**Protein production and IMAC purification.** Chemically competent *E. coli* Rosetta (DE3) cells were transformed with 1 µl of purified plasmid, plated on LB agar plates with kanamycin (50 µg/ml) and chloramphenicol (25 µg/ml), and grown at 37˚C overnight. For each strain, six colonies were used to inoculate six wells of a deep 24-well plate containing LB (6 ml) plus kanamycin (50 µg/ml) and chloramphenicol (25 µg/ml), and the plate was shaken at 37˚C, 220 rpm overnight. Wells were pooled and the mixture (5 ml) was used to inoculate an LB culture (1 L) containing kanamycin (50 µg/ml) and chloramphenicol (25 µg/ml) in an Erlenmyer flask (2.8 L). The culture was shaken at 37˚C, 200 rpm until an OD_600_ of 0.4-0.7 was reached, upon which the cultures were chilled (18˚C for *Ec*LdcA, 15˚C for *Ap*LdcA), induced with isopropyl β-d-1-thioglactopyranoside (IPTG; 1 mM for *Ec*LdcA and 0.1 mM for *Ap*LdcA), and were shaken overnight. Cells were harvested by centrifugation at 4000 rpm, 4˚C for 30 min and the pellets frozen at -80˚C until purification.

To purify proteins, cell pellets (from 500 ml culture) were thawed on ice and suspended in lysis buffer (35 ml; 0.5 M NaCl, 25 mM imidazole, 10% glycerol, pH 8.0, with freshly added 0.4 mg/ml HEWL, 1 mM PMSF, 1 mM MgCl_2_, and 10 µg/ml DNaseI). Cell suspensions were rocked at 4˚C for 1 hour, vortexed, and homogenized using a EmulsiFlex^®^-C3 high-pressure homogenizer. Lysates were centrifuged at 13,000 rpm, 4˚C for 30 min and the supernatants decanted onto Ni-NTA resin (2ml; Qiagen) pre-equilibrated with lysis buffer. The lysate-resin slurry was gently mixed and the flow-through eluted. The resin was washed twice with lysis buffer (25 ml), twice with elution buffer (25 ml; 0.1 M NaCl, 5 mM HEPES, 25 mM imidazole, 10% glycerol, pH 8.0), and once with each of the following elution buffers of increasing imidazole: 50 mM, 100 mM, 200 mM, and 500 mM imidazole (5 ml). Fractions were analyzed by SDS-PAGE (Figure S1C-D), and those containing proteins of interest were pooled, packed into 3500 MWCO SnakeSkin Dialysis Tubing (Thermo), and dialyzed at 4˚C over the course of two days with four exchanges of dialysis buffer (1 L; 0.1 M NaCl, 25 mM Tris, 10% glycerol, pH 7.5, with 5 mM BME added to the first two exchanges). Dialyzed proteins were concentrated using 10 kDa MWCO Amicon Ultra-15 centrifugal filters (EMD Millipore). Protein concentrations were determined from UV absorbance (λ = 280 nm) measured on a NanoDrop Lite spectrophotometer (Thermo) using fully reduced extinction coefficients calculated using the Expasy ProtParam online tool (Swiss Institute of Bioinformatics).

***E. coli* muropeptide-based enzyme assays.** All enzymes used were either purified in or exchanged into Tris buffer (20 mM Tris, 0.1 M NaCl, pH 7.5) prior to treating cell walls. The sacculus from DH5α *E. coli* (0.5 mg) was digested with mutanolysin (2 µM final concentration, Sigma-Aldrich) in Tris buffer (50 µl total volume) and incubated overnight at 37˚C in a thermocycler with the lid heated to 60˚C. Enough mutanolysin reactions were prepared for each downstream LdcA reaction to be run in triplicate. Reactions were heat killed at 100˚C for 15 min and transferred to empty Mini Bio-Spin Chromatography Columns (Bio-Rad) to remove insoluble material by centrifugation for 1 min at 3000 rpm and room temperature. Borate buffer (25 µl; 0.5 M) and < 1 mg sodium borohydride were added to the column flow-through to reduce carbohydrate reducing ends (2). Reactions were allowed to proceed in open air for 30 min before neutralizing with phorphoric acid (6-9 µl; 10%) to pH = 3-4. Column flow-throughs containing soluble muropeptides were desalted via HPLC using the same method described for the purification of muropeptides from aphid homogenate. Collected fractions were dried to completion by speedvac and frozen at -20˚C. The soluble muropeptides obtained through this process were then incubated with LdcA enzyme (2 µM final concentration) in Tris buffer overnight as described for mutanolysin digests, then heat killed and desalted by HPLC.

**Proteomics-based muropeptide analysis.** Each dry sample was resuspended in deionized water (300 µl for enzyme assays, 500 µl for aphid muropeptide fractions), diluted five-fold, and subjected to LC-MS/MS analysis using the same instrument described for proteomic analysis. The enzyme assay sample order was semi-randomized, with multiple blank runs included and no two samples of the same treatment run adjacently. The six aphid PGN fractions were run immediately following the last enzyme assay sample. A 5-µl injection volume was used for each sample. Liquid chromatography of muropeptides followed the methods of Bern *et al.* (3).

Samples were loaded onto the trap column (Acclaim PepMap 100 C18 Trap Column, 2 cm × 75 μm I.D., 3 μm particle size; Thermo) at 4 μl/min with buffer consisting of 0.1% formic acid in water, then switched in line with the analytical column, a PicoFrit Hypersil GOLD aQ C18 column (1.9 µm, 175 Å, 75 µm I.D. × 50 mm; New Objective), 5 min post injection. The chromatographic separation was achieved using buffers A (0.1% formic acid in water) and B (0.1% formic acid in acetonitrile) flowing at 300 nl/min. The gradient began with constant flow of 0.8% B from 0 to 10 min, then increased linearly to 22.5% B by 60 minutes. An MS scan range from *m/z* 300-1600 was used with AGC (automatic gain control) target of 4e5 ions and 50 ms maximum inject time, with ions filtered through the quadrupole and separated in the orbitrap detector with a resolution of 120,000. MS/MS spectra were acquired over a 3-sec cycle time with four MS/MS fragmentation scan modes: EThcD (electron-transfer/higher-energy collisional dissociation) in the orbitrap using priority selection of highest charge states and lowest *m/z* with 60,000 resolution, supplemental activation collision energy at 15%, a maximum inject time of 118 ms, and AGC target of 5e4 ions; HCD (higher-energy collisional dissociation) in the orbitrap with 60,000 resolution, collision energy at 25%; EThcD and ETciD (electron-transfer/collision-induced dissociation) in the ion-trap detector, both with supplemental activation collision energy at 15%, maximum inject time of 35 ms, and an AGC target of 1e4 ions. MS/MS were collected for precursor ions exceeding the threshold of 5e4 ions with charge states of 2-6, where dynamic exclusion was used for 30 sec after 1 scan with an *m/z* range of 120-2000. Calibrated charge dependent ETD parameters mode were selected to determine the optimal reaction time during ETD scans.

Individual PGN compounds were identified from MS and MS/MS spectra using Byonic software (Protein Metrics; version 3.10.10). For each raw LC-MS/MS data file, five separate Byonic searches were performed using different protein database files containing either canonical non-crosslinked, singly crosslinked, or doubly crosslinked sequences, or noncanonical non-crosslinked or singly crosslinked sequences. A fixed modification of +72.0848 was assigned to the amino-acid letter code J as a stand-in for *m*Dap (mass = 172.0848). Muropeptide dimers and trimers were represented by concatenated peptide sequences (e.g. AEJAAEJA for tetra-tetrapeptide dimers). Glycan modifications included GlcNAc-MurNAc (GM; +480.1954) and GlcNAc-anhydro-MurNAc (GaM; +460.1693), with each designated as common at only N- termini for monomers, N- and/or C-termini for dimers, and at J, N- and/or C-termini for trimers. For data generated from samples that did not undergo reduction with sodium borohydride, nonreduced GlcNAc-MurNAc (GM-nonreduced; +478.1799) was also included as a possible modification. Mass tolerances were set at at 5 ppm for prescursors, 20 ppm for FTMS fragments (HCD spectra), and 0.5 Da for ion trap fragments (ETD and EThcD spectra), and a manual score cutoff of zero.

Byonic search hits were manually validated and quantified using Byologic software (Protein Metrics; version 3.10.52). To maintain organization of data, three separate Byologic projects were created: one containing all canonical stem peptide sequences, a second containing noncanonical stem peptides, and a third containing non-reduced muropeptides applicable only to aphid homogenate samples and blanks. For each Byologic project, the “Add Missing Via Existing Peptides” function was applied to obtain extracted-ion chromatograms (XICs) for parent ions that were missing for samples that lacked MS/MS data for those ions. In this way, an XIC was available for each sample in every hit, regardless of whether MS/MS data was present. While most of these added XICs showed no obvious peaks, some contained peaks consistent in shape and retention time with those from other samples that could be validated based on the presence of high-quality MS/MS spectra. Each sample XIC was manually inspected for the presence, shape, and retention time of all peaks, as well as the abundance and score of MS/MS spectra per peak. Hits with inconsistencies in some or all of these were designated as “False-Positive”. We found that samples run early in the LC-MS/MS sequence exhibited earlier retention times than those run later. To account for this, a single integration range was used for every XIC within a hit, extending from the peak retention time of the earliest-run sample to that of the latest. Thus, all XICs were included in all downstream analyses, with the XIC integration area (AUC) taken as a measure of compound abundance per sample.

Because our Byonic parameters allowed for some flexibility in the location and identity of glycan substitutions, it was also necessary to manually identify hits that were labeled as distinct compounds but in reality could not be distinguished from one another. For example, glycans were allowed at either N- or C-termini for dimers, and samples were assigned randomly to one of either the N- or C-terminally substituted hit—we arbitrarily selected samples from only one hit to integrate, but examined MS/MS data from both hits to validate the data. The duplicate hit was then labeled “NoInt” in the Comment column. Consequently, crosslinked compounds are distinguished by the number of each glycan type instead of which stem peptide they are connected to (Figures 2, 3, S1, Table S1). Similarly, some peptide sequences are equivalent in mass (AEJI and AEJL, AEJN and AEJGG, AEJQ and AEJAG, AEJ-AEJA and AEJA-AEJ, AEJ-AEJ(Q/AG) and AEJA-AEJG). For these, we attempted to resolve the peptide identity from their MS/MS spectra, but in most cases this was not possible. Thus, in each resulting figure, multiple compounds are possible for these sequences (Figures 2, 3, S1, Table S1). All raw MS data and both Byonic and Byologic parameter and output files are available for download via the mass spectrometry database MassIVE (MSV000087634).

For each Byologic project, the peptide list was filtered to remove hits designated as “False-positive”, which mainly included hits containing GlcNAc-MurNAc within non-reduced samples, and those labeled as “NoInt”, which mainly included equivalent hits. Lists were exported from Byologic as comma separated files (csv) and imported into RStudio version 1.1.463 (4) running R version 3.6.1 (5). Data transformation, statistical comparison, and plotting were accomplished using custom R scripts written using functions from the tidyverse package (6). To transform the raw data, the XIC AUC values of previously diluted samples were multiplied by five. Baseline subtraction was performed on all samples for each parent mass by subtracting the mean XIC AUC of five blank samples. Data were normalized by dividing each XIC AUC value by the sum of all XIC AUC values per sample. In this way, the normalized or relative XIC AUC value of each compound represents the proportion of total PGN for a given sample, allowing PGN composition to be compared between samples with vastly different PGN abundances, as in Figures 2, 3, S1, and Table S1. Finally, both composition and assay data were log transformed.

Statistical differences among enzyme assay samples were identified by applying Tukey’s Honest Significant Difference (HSD) test on an Analysis of Variance model of the *E. coli* muropeptide dataset (Figure 3, S1E-G). The resulting p-values were adjusted for false-discovery rate via multiple test correction. Plots were generated using the ggplot2 package (7) and brackets indicating statistical significance between two treatments (corrected p-values < 0.05) added using the geom_signif function from the ggpubr package (8). All custom scripts, raw and transformed data, and statistical analyses are available for download at GitHub (https://github.com/smit4227/ApLdcA_proteomics).

**Single-substrate enzyme assays by UPLC-MS.** Enzymes were further purified prior to performing assays with single substrates. Both *Ec*LdcA and *Ap*LdcA, affinity-purified from 1 L and 11 L of culture, respectively, were subjected to FPLC using an ÄKTA pure instrument (GE Life Sciences) equipped with a Superdex 200 Increase 10/300 GL size-exclusion column (GE Life Sciences). The column was pre-equilibrated with two column volumes of running buffer (25 mM Tris, 0.1 M NaCl, pH 7.5) at 4˚C before the concentrated sample was injected (0.5 ml) and 1.5 column volumes eluted at 0.5 ml/min. Seventy fractions were collected (0.5 ml each) for each protein concentrate injected. Selected fractions were subjected to SDS-PAGE to assess purity. Multiple fractions were pooled together for each enzyme, with protein yields of 3.49 mg for and 1.58 mg for *Ap*LdcA. Pooled fractions were buffer-exchanged (25 mM Tris, 0.1 M NaCl, 10% glycerol, pH 7.5) at 4˚C and concentrated using centrifugal filters, then aliquoted and stored at -80˚C.

Synthetic PGNs substrates used in this study are shown in Figure S2 and were synthesized using methodologies previously reported by our laboratories (9, 10). A muropeptide mixture **s12** was purified from mutanolysin-derived *E. coli* muropeptides by HPLC. Collected fractions were concentrated to dryness by speedvac and were reconstituted in reaction buffer (20 mM Tris, 0.1 M NaCl, pH 7.5). Substrate **s12** contains **s12a** (tetra-tri dimer with 4,3-crosslink), **s12b** (tri-tetra dimer with 3,3-crosslink), and **s12c** (tetra-tetra dimer with 4,3-crosslink) in a ratio of ~1:1:5.

Reactions of LdcA enzymes with eleven synthetic PGNs (**s1**-**s11**) were carried out in Tris buffer (20 mM Tris, 0.1 M NaCl, pH 7.5) at room temperature. At each time point (2 h, 8 h, and 24 h for slow turnover substrates), trifluoroacetic acid was added to the reaction mixture to stop reactions. Reaction mixtures were analyzed by UPLC/MS using a Bruker micrOTOF-QII quadrupole time-of flight hybrid mass spectrometer equipped with an Ultimate 3000 RSLC UPLC and controlled by HyStar version 3.2 SR4. LC separations were performed on an Acquity UPLC HSS T3 column (Waters; 2.1 × 150 m, 1.7 μm) at a flow rate of 0.4 mL/min with variations of a 45-min gradient starting from 0% B to 20% B (A = 0.1% formic acid in water, B = 0.1% formic acid in ACN). Detailed LC-MS parameters are reported by Lee *et al.* (10). Reactions of LdcA enzymes with **s12** were carried under the same conditions except that after enzymatic reaction, the mixtures were reduced by sodium borohydride, as described earlier for mutanolysin-derived *E. coli* muropeptides. Reaction outcomes of single-substrate assay are summarized in Table 1 and Figures 4-5, S3-S6.

**References**

1. S. M. Desmarais, F. Cava, M. A. de Pedro, K. C. Huang, Isolation and preparation of bacterial cell walls for compositional analysis by ultra performance liquid chromatography. *J. Vis. Exp.*, e51183 (2014).

2. B. Glauner, Separation and quantification of muropeptides with high-performance liquid chromatography. *Anal. Biochem.* **172**, 451–464 (1988).

3. M. Bern, R. Beniston, S. Mesnage, Towards an automated analysis of bacterial peptidoglycan structure. *Anal. Bioanal. Chem.* **409**, 551–560 (2017).

4. RStudio Team, *RStudio: Integrated development for R.* (RStudio, PBC, 2020).

5. R Core Team, *R: A language and environment for statistical computing.* (R Foundation for Stistical Computing, 2019).

6. H. Wickham, *et al.*, Welcome to the Tidyverse. *J. Open Source Softw.* **4**, 1686 (2019).

7. H. Wickham, *ggplot2: Elegant graphics for data analysis* (Springer-Verlag, 2016).

8. A. Kassambara, *ggpubr: “ggplot2” based publication ready plots* (2020).

9. W. Zhang, *et al.*, Reactions of the three AmpD enzymes of *Pseudomonas aeruginosa*. *J. Am. Chem. Soc.* **135**, 4950–4953 (2013).

10. M. Lee, *et al.*, Catalytic spectrum of the penicillin-binding protein 4 of *Pseudomonas aeruginosa*, a nexus for the induction of β-lactam antibiotic resistance. *J. Am. Chem. Soc.* **137**, 190–200 (2015).
