# Supplementary material for: Horizontal-Acquisition of a Promiscuous Peptidoglycan-Recycling Enzyme Enables Aphids To Influence Symbiont Cell Wall Metabolism
Source: mBio. 2021 Dec 21;12(6):e02636-21. doi: 10.1128/mBio.02636-21 (PMC8689515; doi:10.1128/mBio.02636-21)
Supplement: TABLE S2 [file mbio.02636-21-st002.docx]

| Primer | Description | Tm (˚C) | Sequence (5’-3’) |
| --- | --- | --- | --- |
| 1 | *Ap*LdcA-fwd | 64 | CGCGGCAGC**CATATG**AGCGGCGCAGCAGT |
| 2 | *Ap*LdcA-rvs | 65 | GTGGTGGTG**CTCGAG**CTAAGCTGCTTGGTCAAGCGGTAC |
| 3 | *Ec*LdcA-fwd | 56 | CGCGGCAGC**CATATG**TCTCTGTTTCACTTAATTGCC |
| 4 | *Ec*LdcA-rvs | 54 | GTGGTGGTG**CTCGAG**TTACATTTTAAGAACAGGATGAC |
| 5 | T7 | 48 | TAATACGACTCACTATAGGG |
| 6 | T7term | 55 | GCTAGTTATTGCTCAGCGG |
